# Unravelling Guest Dynamics in Crystalline Molecular Organics Using ^2^H Solid-State NMR and Molecular Dynamics Simulation

**DOI:** 10.1021/jacs.4c03246

**Published:** 2024-06-27

**Authors:** Valentina Erastova, Ivana R. Evans, William N. Glossop, Songül Guryel, Paul Hodgkinson, Hannah E. Kerr, Vasily S. Oganesyan, Lorna K. Softley, Helen M. Wickins, Mark R. Wilson

**Affiliations:** † Department of Chemistry, Durham University, Stockton Road, Durham DH1 3LE, U.K.; ‡ Department of Chemistry, University of Edinburgh, Joseph Black Building, David Brewster Road, Edinburgh EH9 3FJ, U.K.; § School of Chemistry, University of East Anglia, Norwich NR4 7TJ, U.K.

## Abstract

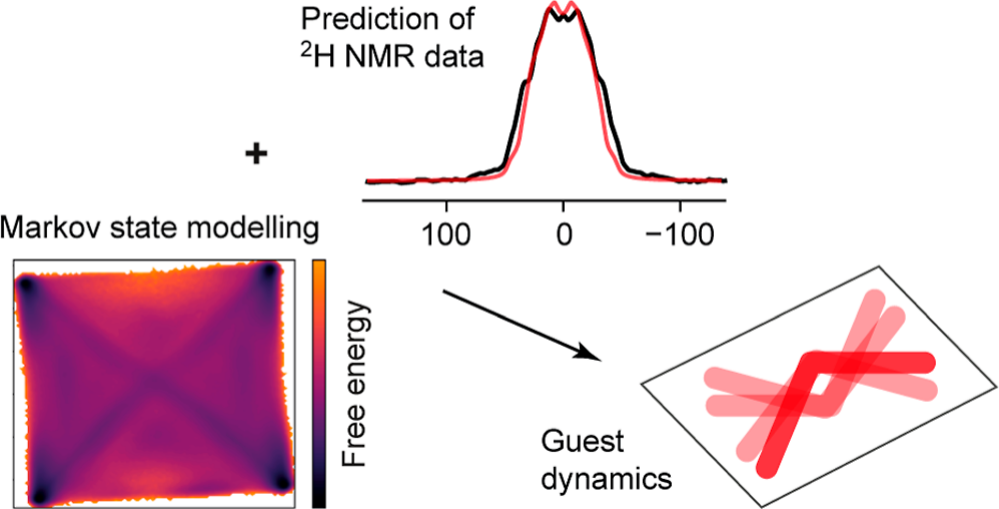

^2^H solid-state NMR and atomistic molecular dynamics (MD) simulations are used to understand the disorder of guest solvent molecules in two cocrystal solvates of the pharmaceutical furosemide. Traditional approaches to interpreting the NMR data fail to provide a coherent model of molecular behavior and indeed give misleading kinetic data. In contrast, the direct prediction of the NMR properties from MD simulation trajectories allows the NMR data to be correctly interpreted in terms of combined jump-type and libration-type motions. Time-independent component analysis of the MD trajectories provides additional insights, particularly for motions that are invisible to NMR. This allows a coherent picture of the dynamics of molecules restricted in molecular-sized cavities to be determined.

## 1 Introduction

Molecular materials often contain disorder, and this disorder may contribute significantly to the relative free energies of different solid forms at ambient temperatures. In the context of pharmaceutical materials, disorder is generally perceived as a risk factor as it is assumed to reflect metastability with respect to some fully ordered structure. Hence, reducing disorder, e.g., via cocrystallization, is generally perceived as desirable.^1^

Disorder is especially common in solvate forms, which are commonly encountered in pharmaceutical chemistry, for example, it has been estimated that up to a third of all active pharmaceutical ingredients (APIs) can form hydrates.^2^ Solvates are frequently encountered when the host crystal lattice packing is relatively inefficient, resulting in channels or voids in the structure that can be filled by small molecules, reducing the free energy of the crystal compared to the nonsolvated form.^3^ Moreover, the solvent is frequently dynamic, potentially providing entropic stabilization. Hence, disorder may be intrinsic to the stability of the phase and is not necessarily a marker of instability. Even if solvate forms are not the final API, they are frequently encountered during pharmaceutical production, and so understanding such forms is important for the characterization of a drug substance and its production.

Characterizing the behavior of disordered guest solvent is often challenging. Bragg scattering is disrupted by dynamic disorder, and solvent molecules commonly appear in diffraction-derived structures as ill-defined volumes of electron density. NMR provides an alternative and often more direct route to characterizing disordered materials,^1,4,5^ including identifying and studying the behavior of solvent molecules in a host crystal structure. Deuterium (^2^H) NMR is particularly useful since it is straightforward and inexpensive to introduce ^2^H isotopic labels using deuterated solvents, and the ^2^H NMR parameters and spectra are affected by dynamic processes on a broad range of time scales.^6,7^ Dynamics on a similar rate to the width of ^2^H NMR spectra (10–100s kHz) results in changes to the ^2^H NMR spectrum line shape, and spin–lattice (*T*_1_) relaxation times are sensitive for faster dynamics (on the order of the ^2^H NMR frequency, typically 10s MHz). Slow motions, of the order of 10s kHz, can also be observed via the widths of sidebands in magic-angle spinning spectra, as has been applied to a variety of systems, including pharmaceutical solvates^8^ and lipid membranes.^9^ In all cases, however, the NMR data cannot be “inverted” to determine the molecular motions involved. Relaxation data are particularly difficult to interpret, and even apparently distinctive changes in ^2^H spectral line shape can lead to oversimplified models of the molecular behavior (as discussed below). Hence, it is desirable to use computational chemistry methods to predict molecular behavior and to link theory and experiment without needing to postulate motional models.

In contrast to other domains, such as biomolecular systems, molecular dynamics (MD) simulations have been used relatively sparingly in solid systems. Although there was some skepticism that atomistic force fields would reproduce experimental behavior in crystalline materials,^10^ there are now many successful examples of the application of MD to solids containing disorder. For example, MD has been applied to glassy molecular solids, showing that well-chosen force fields can accurately reproduce experimental ^13^C NMR shift distributions.^11,12^ Similarly, averaged NMR parameters derived from MD simulation have been used to understand the dynamics of molecules on catalytic surfaces,^13,14^ lipid membranes,^15,16^ and ionic liquids.^17^ MD simulation has also been used to predict NMR relaxation time constants and probe the contribution of different motions in lipid bilayers.^18,19^ Most applications to highly crystalline systems have involved porous or channel-containing host–guest systems. For example, the fast rotational and translational dynamics of urea inclusion systems can be effectively studied by conventional atomistic MD, see ref (20) and references therein. Studies of gas absorption and diffusion in metal–organic frameworks use different approaches to simulation, notably Grand Canonical Monte Carlo simulations.^21,22^ A variety of methods have been used to study the dynamics of so-called “amphidynamic” crystals,^23−25^ solid systems which contain highly mobile components alongside a rigid molecular framework. Typically, the experimental rate information is derived from NMR, either ^1^H *T*_1_ relaxation rates or ^2^H NMR spectra.^26^ Such materials have been proposed as “molecular machines”, e.g., crystalline molecular gyroscopes,^26^ in which a molecular fragment (a “rotor”) is relatively free to rotate, or molecular gears,^27−29^ in which there is interaction between mobile components. Especially in the latter case, simulations of larger box sizes are needed to understand to the effects of correlation between different rotors.^28−30^ Correlated motions often require techniques such as metadynamics^28,31^ to enhance sampling beyond standard MD and to improve the sampling of dynamical pathways on complex free-energy surfaces.

In the case of “molecular machines”, the motion of the fragment is well-defined, and the challenge is to determine the rate of motion and how this is influenced by molecular interactions. In the case of pharmaceutical solvates, the nature of the guest molecule motion is undefined, and it is not obvious how to use MD trajectories to derive a physical picture of the motion or compare it to the experiment. Dimethyl sulfoxide (DMSO) molecules have been observed to rotate through 180° in MD simulations of a DMSO solvate of carbamazepine, allowing the experimental crystallographic data to be rationalized.^32^ MD simulations have been used in a similar way to rationalize experimental data on the dynamics of pyridinium cations obtained from quasi-elastic neutron scattering^33−35^ and ^19^F NMR relaxation data observing molecular motion in solid octafluoronaphalene.^36^

**Figure 1:**
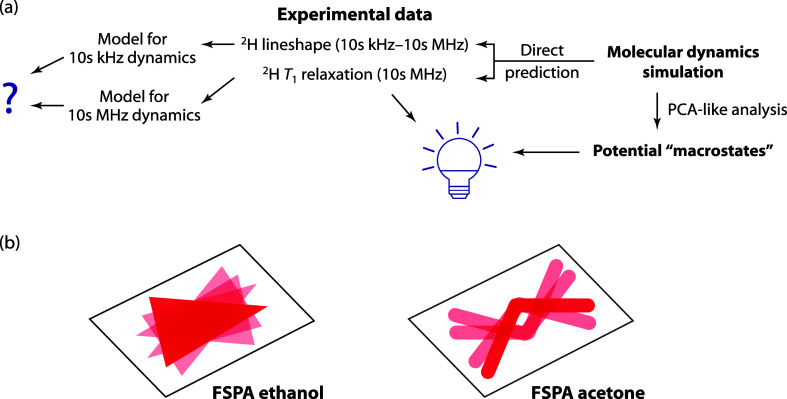
Overview of paper. (a) ^2^H NMR data in the solid state is typically analyzed using distinct models relevant for 10s kHz and 10s MHz dynamics for line shape and relaxation data, respectively. Particularly when the motion is complex, reconciling these pictures can be difficult or impossible. Here, we directly predict line shapes and relaxation data from the MD trajectories, avoiding the troublesome “dissection” of the normal approach. (b) Cartoon representation of the resulting physical picture. Since the voids occupied by the solvent molecules have inversion symmetry, the overall dynamics of the solvent mirrors this symmetry. In both cases, the motion has a strong librational character, but a complementary PCA-like analysis also identifies distinct “macrostates” that reflect the local symmetry of the solvent sites.

So, while MD has been regularly applied to solid crystalline materials and NMR is widely used to obtain information (such as activation barriers) from materials exhibiting dynamics, the general question of how to determine the nature of motion of guest molecules moving in a host cavity has not, to our knowledge, been directly addressed. As illustrated schematically in Figure 1, a typical approach is to fit the NMR data, whether spectral line shapes or relaxation times, to simple motional models. These models are either assumed or obtained qualitatively from the simulation methods. Here, we adapt an approach previously used to predict EPR line shapes directly from the results of MD simulations to ^2^H NMR and show that the simultaneous prediction of ^2^H relaxation and line shape data provides a more complete physical picture than indirect comparisons involving simplified models.

This methodology is illustrated using a previously unreported cocrystal (FSPA) formed between furosemide (FS) and picolinamide (PA). ^2^H solid-state NMR and MD simulations are used to probe the behavior of FSPA with acetone and ethanol as the solvent guest. The direct prediction of the NMR data from MD simulation, combined with a complementary principal-component-type analysis, allows the overall molecular behavior to be understood in terms of the local site symmetry. This methodology can be readily applied to other solid systems in which guest molecules move within cavities.

## 2 Methods

### 2.1 Synthesis and Crystallography

Furosemide is an important pharmaceutical, as listed in the World Health Organization’s List of Essential Medicines, primarily used in relieving fluid accumulation (edema) in the heart, liver, or kidney. Cocrystallization of FS has been widely investigated as a route to improving its relatively poor aqueous kinetic solubility.^37−39^ Under its synthesis conditions, as described in Section 1 of the Supporting Information, the FS PA cocrystal readily forms a solvate phase.

**Figure 2:**
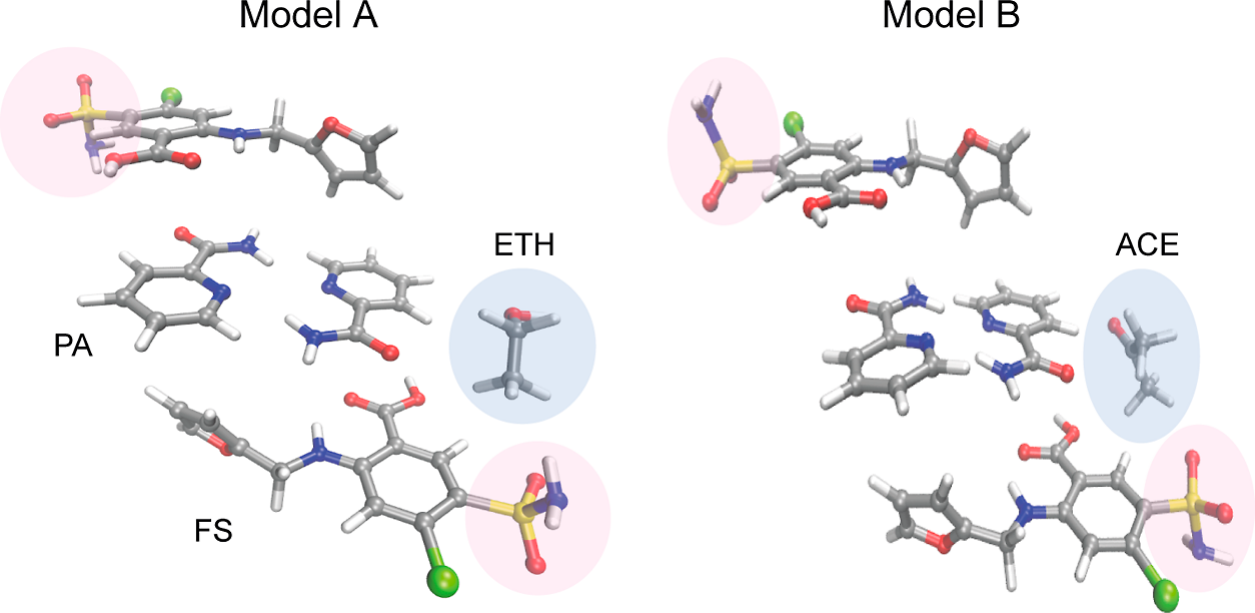
Illustrations of the structure of the ethanol (ETH) and acetone (ACE) furosemide (FS) picolinamide (PA) cocrystal solvates derived from single-crystal XRD experiments at 120 K. Disorder of the sulfonamide group was modeled with a 50:50 split site model, which was resolved into two models, A and B, depending on whether the NH_2_ of the sulfonamide points toward the solvent channel (A) or away from it (B). The volume occupied by the solvent (highlighted in blue) was not modeled in the diffraction study. See Figure S1 in the Supporting Information for an alternative crystallography-oriented depiction of the structure.

Single-crystal diffraction (SCXRD) studies of these materials, discussed in Section 2 of the Supporting Information, reveal that the FSPA acetone and ethanol solvates are isostructural. Fourier difference maps revealed electron density that is separate from the FS and PA framework, which is assumed to correspond to the solvent, but the geometry could not be refined for either solvent molecule. Having confirmed the presence of solvent using solid-state NMR (cf. Figure S2), the SCXRD structures were solved with the PLATON SQUEEZE approach.^40^ As illustrated in Figure 2, the unit cell contains two pairs of symmetry-equivalent FS and PA molecules and a void at an inversion center in which the additional electron density resides, suggestive of a channel solvate. The sulfonamide group is refined with disorder over two positions with (fixed) equal occupancies.

### 2.2 Solid-State NMR

High-resolution solid-state NMR spectra were obtained using either a Bruker Avance III HD spectrometer operating at ^1^H, ^13^C, and ^2^H NMR frequencies of 499.70, 125.65, and 76.71 MHz, respectively, or a Bruker Avance III HD spectrometer operating at the corresponding frequencies of 400.17, 100.62, and 61.42 MHz. Samples were packed into 4 mm zirconia rotors. The ^13^C shift scale was referenced with respect to neat tetramethylsilane (TMS) by setting the highest frequency peak of adamantane to 38.5 ppm. The ^13^C spectra were used to fingerprint the structure and assess stability with respect to solvent loss; see Section 3 of the Supporting Information for further details. The ^2^H shift scale was referenced with respect to neat TMS by setting the peak of D_2_O to 4.81 ppm. Temperatures are quoted to the nearest 5 K to reflect uncertainties in absolute temperature calibration (details of the temperature calibration can be found in the data archive).

Variable-temperature ^2^H wide-line (static) spectra and *T*_1_ relaxation data were acquired at 76.71 MHz for the FSPA-acetone-*d*_6_ sample and 61.42 MHz for the FSPA-ethanol-*d*_2_ sample. FSPA-ethanol-*d*_2_ spectra used 0.2–0.5 s recycle delay and a 40 μs quadrupole echo delay. FSPA-acetone-*d*_6_ spectra used a 1 s recycle delay and a 60 μs quadrupole echo delay. Between 60,000 and 72,000 acquisitions were required to obtained good quality spectra.

^2^H *T*_1_ relaxation measurements were performed by using a saturation recovery sequence. FSPA acetone-*d*_6_ data were measured under static conditions using 16 increments, 64 acquisitions, and a sample maximum recovery time ranging from 1.8–3.2 s, while FSPA ethanol-*d*_2_ was measured under 10 kHz MAS (for improved S/N ratio) using 16 increments, 64 acquisitions, and sample maximum recovery times ranging from 0.26–0.85 s. The spectra were integrated across the complete band shape/sideband pattern. Excellent fits of these integrals were obtained to single exponential decays as a function of recovery time, except for the ethanol solvate at low temperature (see the later discussion). These single-exponential fits are consistent with a high degree of solvent disorder. The temperature dependence of the relaxation times was fitted assuming a simple Arrhenius-like dependence of the motional correlation time, τ_c_, on temperature. The temperature dependence of τ_c_ was parametrized using the parameters, *T*_min_ and *E*_a,_
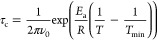
1where *v*_0_ is the ^2^H NMR frequency. As discussed in Section 4 of the Supporting Information, this parametrization reduces the correlation between fitted variables and allows uncertainties to be propagated without the need for Monte Carlo simulations. ^13^C *T*_1_ relaxation times were measured on FSPA ethanol under MAS conditions, but the data were significantly lower quality and are not considered further.

### 2.3 MD Simulations

Simulation models for the two FSPA solvates were built using the atomic coordinates and unit cell parameters obtained by XRD at 120 K (corresponding CIF files are supplied in the data archive). An ethanol or acetone solvent molecule was manually placed in the void in the FSPA unit cell (i.e., modeling 100% solvent occupancy), and simulation boxes were made up of 9 × 3 × 3 unit cells and contained 162 FS, 162 PA, and 81 solvent molecules. To assess the importance of the sulfonamide disorder depicted in Figure 2, pairs of simulations were created for both systems, containing uniquely one orientation or the other, with the NH_2_ of the sulfonamide either facing pointing toward the solvent channel (model A) or away from it (model B). Although the physical system will contain a random distribution of sulfonamide orientations, these artificially fully ordered systems can be expected to span the range of possible solvent dynamics.

MD simulations were performed with the GROMACS 2016.4 suite,^41,42^ using the GAFF force field^43^ obtained from the AmberTools 18 package with the AM1-BCC charge model.^44^ Each simulation was first energy minimized using the steepest descent algorithm, with a convergence criterion of 500 kJ mol^–1^. FS and PA molecules were then positionally restrained using the LINCS algorithm, while the solvent molecules were allowed to move freely and relax into equilibrium positions. The system was pre-equilibrated for 1 ns in the *NPT* ensemble with the velocity-rescale Berendsen thermostat at 120 K, with a temperature coupling constant of 0.1 ps and an anisotropic Berendsen barostat applied with a reference pressure of 1 bar and a pressure coupling constant of 20 ps. The positional restraints on the FS and PA molecules were then removed after the solvent molecule relaxation, and the whole system was allowed to equilibrate for an additional 1 ns with the same protocol as above, with the temperature set to 120 K, as in the original crystal structure determination.

This initial equilibration was followed by an annealing run heating the system from 120 to 350 K, in steps of 30 or 50 K over 10 ns, followed by 10 ns relaxation to prevent a hysteresis before the next temperature increment. H-heteroatom bonds were constrained using the LINCS algorithm, with other parameters as above. Structures were extracted at the temperatures of 150, 200, 250, 300, and 350 K, and then simulated in the *NPT* ensemble with the Nosé–Hoover thermostat at the given temperature with a temperature coupling constant of 1 ps and an anisotropic Parrinello–Rahman barostat employed at 1 bar, with a pressure coupling constant of 20 ps. Production simulation runs were performed for 200 ns without bond constraints. The 150 K simulation was extended to 400 ns for improved sampling statistics.

The experimental and predicted densities at 120 K are in reasonably good agreement (3–5% deviation from Table S8). The deviations could result from several factors, including experimental uncertainties in the exact temperature of the crystal in the SCXRD measurements and nonstoichiometry of the solvent loading (i.e., less than 100% occupancy of the solvent voids). We argue below that reproduction of the experimental NMR data provides a more direct measure of the effectiveness of the force field performance.

The resulting trajectories were analyzed using two approaches: direct prediction of the NMR results from the trajectories (described below) and a Markov State Modeling (MSM). The MSM analysis was performed using the PyEMMA 3.5.4 package.^45^ Two vectors describing each solvent molecule were extracted from the MD trajectories and used for featurization. This approach offers a dimensionality reduction by removing unnecessary atomic coordinates and significantly speeds up data processing.^46^ The time-lagged independent component analysis method was used to decouple any fast vibration-like motions from the significant solvent reorientations.^47^ The resulting time-independent components (TICs) were then used to derive Markov state models, using *k*-means clustering to discretize the data to a representative set of microstates. An appropriate MSM lag time was determined to be 5 fs (10 timesteps) for both the acetone and ethanol systems based on implied time scales plots. Perron-cluster–cluster analysis (PCCA+) was used to cluster the microstates into a limited number of macrostates.^48^ Chapman–Kolmogrov tests^49^ were used to validate the resulting Markov state models, and transition-path theory was applied to determine the flux between macrostates;^50−52^ see Section 7 of the Supporting Information for further details.

### 2.4 Prediction of NMR Spectra from MD Simulations

The simulation methodology developed previously for predicting EPR spectra of molecular systems with introduced spin labels and probes from MD trajectories^53,54^ has been adapted for the simulation of ^2^H NMR line shapes. The MD-EPR methodology has been successfully applied to various complex molecular systems, including proteins, liquid crystals, lyotropic mesophases, lipid membranes, and DNA fragments.^55−59^ Generally, dynamic problems in magnetic resonance can be expressed in terms of the Stochastic Liouville Equation^54^
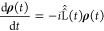
2where **ρ** is a density matrix of the system and the Liouvillian, , is a superoperator of the interaction Hamiltonian.

Equation 2 can always be stepwise integrated over sufficiently short incremental time steps to capture the evolution of the NMR spins due to the molecular dynamics. In practice, this is very inefficient, and assumptions about the time scales of motions are required. A common assumption in ^2^H NMR is that changes in spectral line shape reflect dynamic processes on the same frequency scale as the width of the ^2^H spectrum (typically a few 100 kHz). A connected assumption is that dynamics on this relatively slow time scale involve stochastic jumps between a limited number of Markov states. This corresponds to expressing  in eq 2 in terms of the diagonal matrix whose elements are the precession frequencies of each state and a kinetic matrix containing the jump rates between the states.^60^

Alternative approaches are required where the dynamics are more complex and cannot be factored into a purely jump model. Here, it is desirable to simulate the spectra directly from explicit MD trajectories. The theory is presented in detail in Section 5 of the Supporting Information, and only an overview is given here. The key concept is that if the MD trajectories are sufficiently long to capture the relevant dynamic process, then the solution of eq 2 in the fast motional regime can be written in the form^53,54^
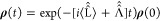
3where  is a Liouvillian which is averaged over the time of complete relaxation of the correlation function of the molecular motion (*T̃* ≈ 10 τ_c_) and over the *N* copies of the molecules in the simulation; this describes the “average” evolution.

The other term in eq 3 describes the line-broadening effects of the dynamics, which is captured by the “decoherence matrix” .  defines the dephasing of the magnetization caused by the modulation of  due to the reorientational dynamics.

There are two advantages of using eq 3 over direct integration of eq 2. First, it offers a significant reduction in the overall simulation time compared to full propagation of the density matrix along the entire sampling time required for the desired resolution in the NMR spectrum. Second, long sampling times of up to milliseconds are required to accurately simulate ^2^H NMR line shapes, which is impractical for many MD simulations. The use of eq 4 allows predictions of NMR data from relatively short MD trajectories (up to the point when autocorrelation functions of rotational dynamics are fully relaxed).^54^

In practical terms, trajectories from all individual molecules are first concatenated into a single continuous one using the appropriate rotational transformations. Motionally averaged tensors are then calculated, from which average quadrupolar coupling parameters, ⟨χ⟩ and ⟨η⟩, can be determined. These determine the time-averaged Liouvillian, , and the frequencies of the ^2^H transitions. The decay rates of the transitions are determined by the elements of the decoherence matrix  (as described in the Supporting Information). This allows for a spectrum to be calculated for a given crystallite orientation. Finally, the response is averaged by assuming an isotropic distribution of crystallites in the sample.

The only inputs to the calculation other than MD trajectories are estimates for the quadrupolar coupling in the absence of dynamics. The χ quadrupolar parameter for FSPA ethanol was taken from a fit of the low-temperature experimental data, as shown in Figure S3. The corresponding values for the methyl group of the acetone solvate were taken from the literature,^61^ with the effect of rapid rotational diffusion accounted for by applying eq S14, with the angles α = 0, β = 70.50°, resulting in the values χ = 53.25 kHz, η = 0.

### 2.5 Prediction of *T*_1_ Relaxation Times from MD Simulations

The longitudinal relaxation times *T*_1_ of ^2^H due to quadrupolar interactions under the Redfield approximation and in the case of restricted local molecular motions are given by the standard expression^62,63^
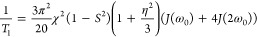
4where *S*^2^ is the square of the generalized order parameter of the molecular orientations, and *J*(ω) is the spectral density function, which is the one-sided Fourier transform of the overall normalized reorientational correlation function
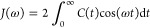
5and ω_0_ is the NMR frequency (expressed as an angular frequency). As discussed in more detail in the Supporting Information, the overall correlation function in solid systems is
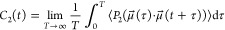
6where *P*_2_(*t*) is a second-order Legendre polynomial and the average is taken over MD simulation time and the number of molecules in the system.  is the unit vector along one of either the equivalent C–D or C–CD_3_ axes in ethanol and acetone molecules, respectively (rotational diffusion about the C–CD_3_ axes is much faster than the reorientational processes of interest).

To interpret the calculated *T*_1_ values, it is also useful to calculate an effective correlation time of the reorientational motion from *C*_2_(*t*) using the expression
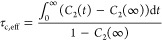
7

A corresponding generalized order parameter can be estimated from the correlation function as *S*_2_ = *C*_2_(∞).

In all cases, MD trajectories of 200 ns in length were sufficient to calculate NMR line shapes and *T*_1_ relaxation times, except simulations at *T* = 150 K, where extended trajectories of 400 ns were required to adequately capture the much slower reorientational dynamics of the solvent molecules.

## 3 Results and Discussion

**Figure 3:**
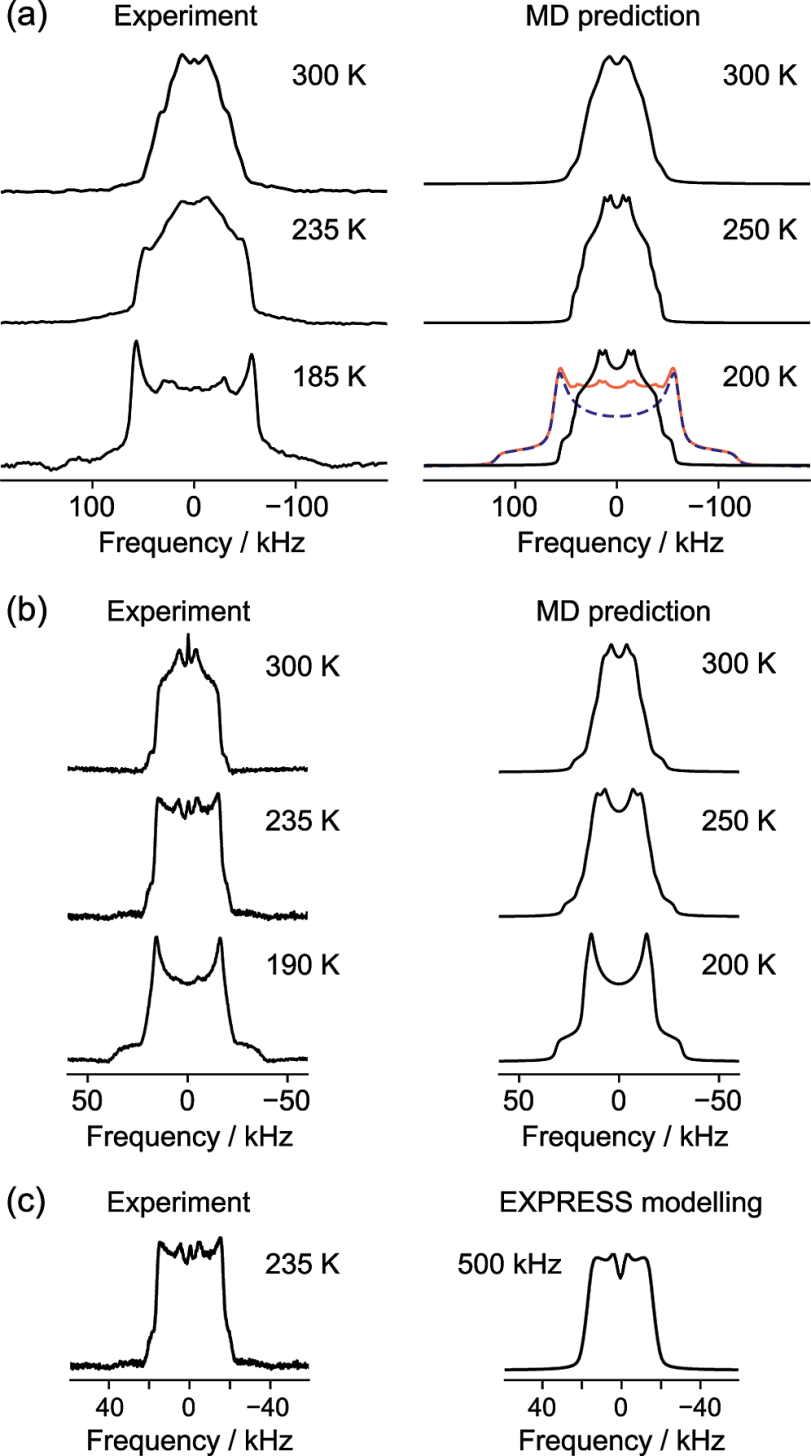
^2^H quadrupolar echo spectra of (a) FSPA ethanol-*d*_2_ and (b) FSPA acetone-*d*_6_. The sharp peak in the center of the spectra in (b) at 235 and 300 K probably reflects trace adventitious acetone. The right-hand sides show MD-predicted spectra at the closest matching temperatures. These are 50:50 sums over models A and B; individual contributions from models A and B are shown in Figures S8 and S9 of the Supporting Information. The experimental spectrum of the ethanol solvate at 185 K contains a significant contribution from the “frozen limit” line shape. The total predicted line shape at 200 K (red) is the sum of 20% dynamic component and 80% static component (dashed blue). (c) Comparison of the 235 K spectrum of (b) with a spectrum simulated with a two-state Markov model.

**Figure 4:**
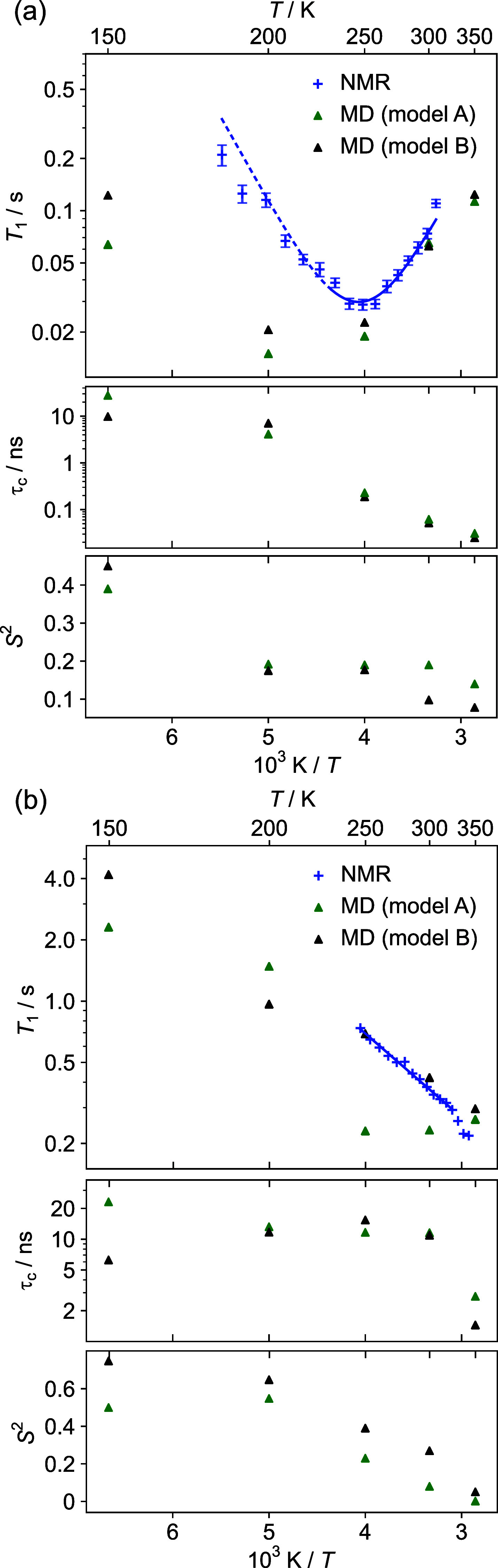
^2^H spin–lattice relaxation time constants as a function of the temperature of (a) FSPA ethanol-d_2_ acquired at 61.42 MHz and (b) FSPA acetone-d_6_ at 76.7 MHz. Triangles are predictions from MD simulation. Error bars (showing one standard error) were obtained from the fitting residuals but are too small to show in (b). Lower panels show τ_c,eff_ and *S*^2^ parameters for the motion derived from MD. The fit to the experimental data in (a) corresponds to *E*_a_ of 19.0 ± 0.9 kJ mol^–1^, although the data are less reliable at low *T* (indicated by dashed line). The apparent fit to Arrhenius behavior in (b) is misleading and cannot be interpreted in terms of *E*_a_ (see the text).

The experimental data presented in Figures 3 and 4 appear sufficiently distinctive to be interpreted; there are significant changes in both ^2^H line shapes (Figure 3) and in *T*_1_ (Figure 4) with temperature. Indeed, as shown in Figure 3c, the spectrum of the acetone solvate can be reproduced by a simple two-state Markov model corresponding to “intermediate time scale” C_2_ jumps about the C=O axis (see Figure S6 of the Supporting Information for further comparisons). As shown by the MD results, however, this naïve model is incorrect, and the apparent jump rate of 500 kHz for the 235 K spectrum is about two orders of magnitude too small.

There are also difficulties in interpreting the *T*_1_ relaxation data, as shown in Figure 4. Although the temperature dependences can be fitted to Arrhenius behavior, it is not obvious that the parameters obtained relate to molecular behavior. For example, the activation energy for the acetone motion of 7.9 ± 0.2 kJ mol^–1^ derived from Arrhenius-type analysis is small and would be expected to correspond to a fast motion, while the sign of the slope implies that the motion is still slow compared to the ^2^H Larmor frequency (10s MHz). This inability to reconcile models of the line shape and relaxation data taken in isolation is illustrated schematically in Figure 1a.

In contrast, the comparison between the experimental ^2^H NMR spectra and those predicted from MD trajectories in Figure 3 is highly revealing. The overall qualitative agreement is excellent, showing both that the MD simulations have captured the solvent behavior, and that the calculation methodology of Section 2.3 is appropriate. Exact quantitative agreement is not expected since there are no adjustable/fitting parameters (in contrast to the modeling shown in Figure 3c), the approximations involved in force-field-based MD, and the experimental uncertainties discussed previously. Note that, as discussed in Section 6 of the Supporting Information, the rotation of sulfonamide groups is slow in comparison to solvent reorientation. Hence, we provide results that are an average of model A and B simulations, noting that the solvent dynamics are sufficiently similar in these idealized models for the overall trends to be validated.

Comparing experimental and MD-predicted spectra of the ethanol solvate at 185 K, Figure 3a reveals that the experimental spectrum contains a significant contribution from the “frozen limit” line shape corresponding to static ethanol molecules. This behavior was reproducible, and similar unexpected contributions from “frozen” guest molecules have been observed in other studies and tentatively rationalized in terms of temperature gradients across the sample.^64^ This explanation seems unlikely here as there is evidence of biexponential behavior in the ^2^H relaxation times, shown by the increased error bars in Figure 4a over a significant range of temperatures (indicated by the dashed fitting line). Example fits are shown in Figure S7 of the Supporting Information. It is, therefore, unsurprising that this temperature regime is also associated with large discrepancies between MD-predicted and experimental *T*_1_ values.

Figures 3 and 4 show that the general trends in the experimental data can be reproduced from the MD simulation, but this does not, in itself, provide insights into the nature of the motion. This can be most obviously obtained by analysis of the MD trajectories themselves (as discussed below), but it is also useful to parametrize the correlation functions derived from the MD trajectories in terms of simple restricted rotational diffusion, involving an effective correlation time, τ_c,eff_, and a generalized order parameter, *S*^2^. Given the complexity of the potential surfaces, the correlation functions are not expected to be simple exponentials (examples are shown in Figure S10 of the Supporting Information), but the general trends in τ_c_ and *S*^2^, as shown in the lower panels of Figure 4 (and tabulated in Table S6 of the Supporting Information), are expected to be robust. Notably, both parameters vary with the temperature. This clearly shows that simple jump-type models, which involve a fixed geometry (amplitude) motion, will not correctly describe the dynamics, even if plausible fits to the experimental data can be obtained (Figure S6). Moreover, the assumption that the correlation time is associated, via an Arrhenius-type relationship, to a thermally activated jump process is clearly incorrect for the acetone dynamics; the τ_c,eff_ values are essentially independent of temperature over the range 200–300 K, rather it is the amplitude of motion (parametrized by *S*^2^) that changes with temperature. This implies that the acetone dynamics has a strong librational character (only the amplitude and not the frequency of motion changes with temperature for barrierless motions).

The fact that the solvent dynamics involves both libration- and jump-type motions is confirmed by comparing the predicted *T*_1_ relaxation times with experimental values, as shown in Figure 4. Recalling that the trends in the experimental data could not be rationalized, the fact that MD simulations correctly predict trends in both ^2^H NMR spectra and relaxation data shows that the simulations are correctly capturing the solvent dynamics. Significantly, this dynamics is consistently on a fast NMR time scale, with motional correlation times measured in ns, and so the traditional approach of analyzing changes in ^2^H spectra in terms of intermediate time scale dynamics, cf. Figure 1a, is destined to give misleading results.

**Figure 5:**
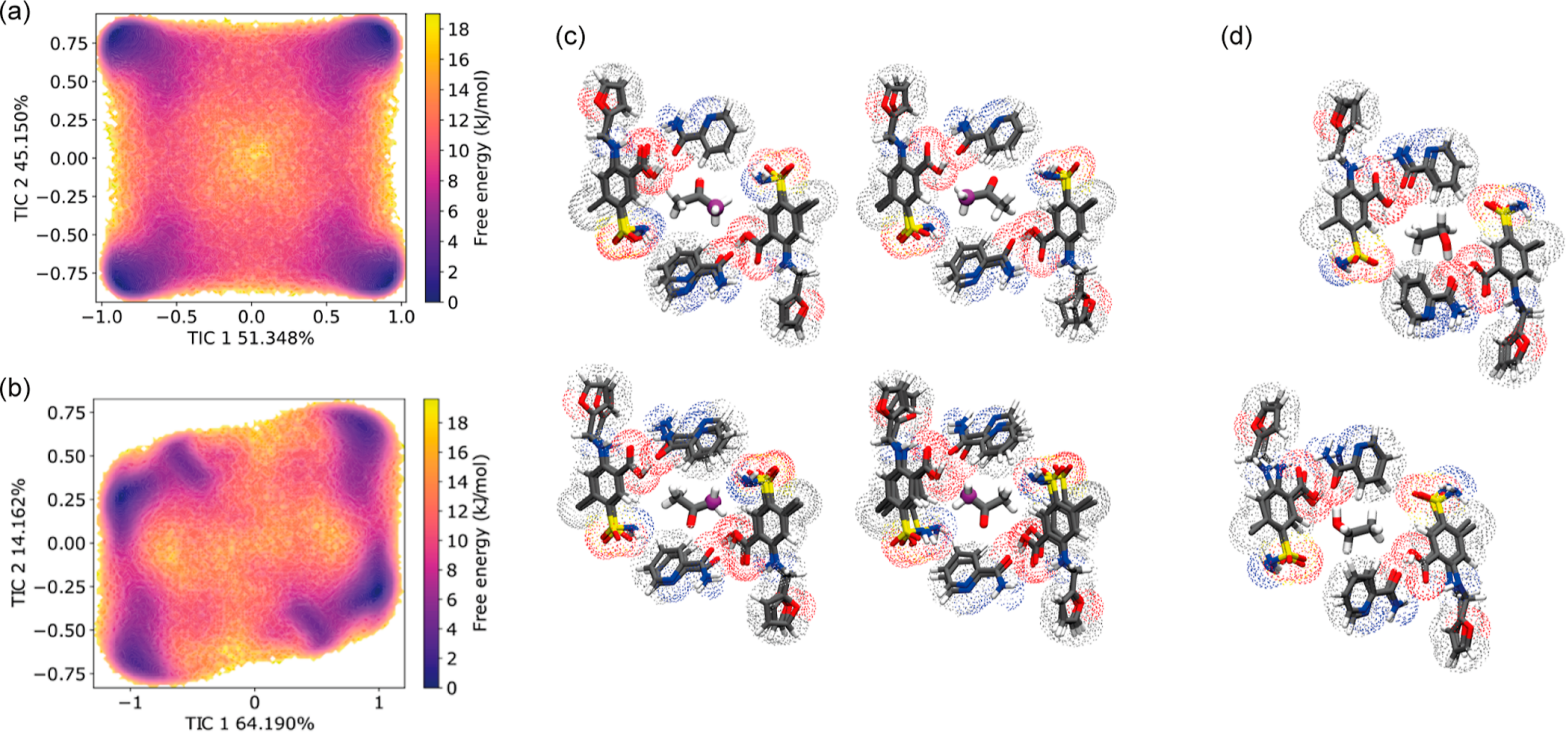
TIC analyses of the FPSA solvate simulations at 273 K. Free-energy plots as a function of the first two TICs for (a) FSPA acetone and (b) FSPA ethanol. (c) Representative snapshots of macrostates corresponding to the four free-energy minima in acetone. One of the methyl carbons of the acetone is colored magenta to highlight the reorientation. (d) Representative snapshots of macrostates corresponding to the two free-energy minima in ethanol.

Additional complementary insight into molecular behavior provided by MSM analysis of the MD trajectories. Figure 5 shows the result of applying the pyEMMA analysis suite to the solvent molecule MD trajectories. In the case of the acetone solvate, two components are identified that account in total for >95% of the variance of the motion. A free energy plot obtained by projecting the trajectories into this space, Figure 5a, shows four distinct minimum energy states. As illustrated in Figure S15 of the Supporting Information, the MD trajectory can be clustered according to these states, allowing representative snapshots, Figure 5c, to be extracted, which correspond to four distinct “orientations” of the acetone within the cavity. These macrostates are related by a *C*_*2*_-axis (respecting the molecular symmetry) and an inversion center (respecting the symmetry of the cavity).

The MSM analysis of the ethanol solvates shows that the ethanol motion is complex, with a higher degree of librational character; only 78% of the variance is captured by the first two TICs. There are two distinct macrostates in the free energy plot, shown in Figure 5b separated by a 180° rotational flip, as shown in Figure 5d. Each of these states contains three sub-states corresponding to rotation about the molecular long axis, with H-bonding occurring in each case between the ethanol hydroxyl group and oxygen H-bond donors of FSPA. See Section 7 of the Supporting Information for the more detailed quantitative analysis of the motion.

## 4 Conclusions

Two analysis approaches have been applied to MD simulation data to understand the dynamics of solvent molecules in cocrystal solvate materials.

A Markov State Modelling analysis, which has previously been applied mainly to protein dynamics, was successful in identifying stable states, pathways between them, and corresponding transition rates. This analysis provided direct insight into slower larger amplitude motions (as distinct from faster motions, such as libration), identifying two main states for ethanol within the FSPA structure and four symmetry-equivalent states for acetone. This two-fold and four-fold degeneracy is consistent with the local molecular and crystallographic symmetry. It is important to note, however, that this insight cannot be obtained from comparing predicted and experimental NMR data, since the ^2^H quadrupolar tensor is invariant under inversion, and so inversion-type dynamics will be invisible to ^2^H NMR. This weakness is shared with classic approaches to modeling ^2^H line shapes using Markov jump-models, where a simplistic model based on C_2_ jumps provides a satisfactory (but ultimately incorrect) fit to the experimental data.

Overall, the solvent disorder is seen to be an intrinsic property of the system that provides some entropic stabilization, precisely because the symmetry of the solvent and the guest cavity do not match. Similar behavior has been observed in the solvates of droperidol, where the adoption of disordered vs ordered structures could be rationalized on simple free energy grounds, rather than any intrinsic metastability of the disordered structure.^65^

Direct simulations of ^2^H NMR spectral line shapes and *T*_1_ relaxation times, using methodology previously applied to the EPR spectra, show that the MD simulations capture the dynamics observed in NMR, and that both the line shape and relaxation can be explained by the same processes occurring on fast (ns) time scales. Crucially, both libration and jump-type motions are treated on an equal footing, avoiding trying to fit line shapes purely in terms of jump-type models. Misleadingly, the experimental spectra can be modeled using simple jump models with μs, despite the molecular motions being on the ns time scale. This explains why conventional approaches to interpreting ^2^H NMR data gave inconsistent and misleading results. It is, therefore, important when dynamic processes are observed via temperature-dependent ^2^H spectra to check whether *T*_1_ relaxation time constants are short, as this would strongly suggest that simple jump models should not be used.

The combination of experiments to provide the overall structure (from SCXRD) and rate information (^2^H NMR), together with the analysis of the MD simulations to provide insight into the molecular motion, leads to a powerful methodology to explain the complex motions often seen in solid materials. Introducing ^2^H site labels is particularly straightforward and inexpensive in host–guest materials, such as solvates or framework materials with organic guests.^66^ We anticipate a more complete understanding of dynamics, especially in more complex systems, e.g., involving coupled rotors,^30^ will be possible by combining sophisticated MD simulation analysis methods and experiment. As well as exploring complex functional materials, robust protocols that confirm the nature and origin of molecular disorder will be important in pharmaceutical applications and in host–guest chemistry more generally. Confirming that a disordered material is at a likely thermodynamic minimum provides increased confidence in its overall stability.

## Supporting Information Available

PDF containing information on: synthesis and crystallography of the FSPA solvates; ^13^C NMR; explanation and justification of parameters used to fit ^2^H relaxation data; details of ^2^H NMR, naïve modeling, and expanded details of direct prediction methodology; additional analysis of MD results, including analysis of the sulfonamide behavior; and further details of the MSM analysis (PDF)

## Notes

Additional research data for this manuscript can be found at http://doi.org/10.15128/r2s4655g65j.
