# Supplementary material for: Unravelling Guest Dynamics in Crystalline Molecular Organics Using 2H Solid-State NMR and Molecular Dynamics Simulation
Source: J Am Chem Soc. 2024 Jun 27;146(27):18360–9. doi: 10.1021/jacs.4c03246 (PMC11240262; doi:10.1021/jacs.4c03246)
Supplement: Supplementary file 1 — ja4c03246_si_001.pdf [file ja4c03246_si_001.pdf]

# Unravelling guest dynamics in crystalline molecular organics using $^2$H solid-state NMR and molecular dynamics simulation: Supplementary Information

Valentina Erastova, Ivana R. Evans, William N. Glossop, Songül Guryel, Paul Hodgkinson, Hannah E. Kerr, Vasily S. Oganesyan, Lorna K. Softley, Helen M. Wickins and Mark R. Wilson

## 1. Synthesis of powder samples of FSPA acetone and ethanol solvates

FSPA acetone and ethanol solvate samples were prepared by liquid-assisted grinding (LAG) of 1:1 molar quantities of the starting materials, 0.165 g furosemide (FS, 0.5 mmol) and 0.061 g picolinamide (PA, 0.5 mmol). For the acetone solvate, FS and PA were ground in 1 ml acetone for 5 mins. A further 1 ml was added and after 5 mins a further 0.5 ml acetone was added every 10 mins until the total LAG time was 30 mins. Powder X-ray diffraction was used to confirm the purity of the products. PXRD measurements were carried out at room temperature using a Bruker AXS d8 Advance diffractometer utilising CuKα radiation and a Lynx-Eye detector. Patterns were recorded in a range of 10° < 2*q* < 60° with a step size of 0.02° and step time of 0.5 s. All patterns were analysed in TOPAS Academic software.[1,2]

A deuterated acetone solvate was produced by the same method using acetone-$d_6$. For the ethanol solvate, the FS and PA components were dry ground for 5 mins before 10 drops of ethanol were added every 5 mins until the total grind time was one hour. A deuterated ethanol solvate was produced by the same method using ethanol-1,1-$d_2$; ethanol-1,1-$d_2$ was chosen over ethanol-$d_5$ to focus on the overall solvent motion. Both syntheses yielded slightly damp cream powders, which were left to dry in air for one hour before packing in 4 mm rotors for subsequent magic-angle spinning (MAS) NMR studies. Samples were stored in the rotor to limit desolvation. $^{13}$C MAS NMR confirmed that the deuterated samples were identical to their non-deuterated analogues.

## 2. Crystallography of FSPA solvates

Single crystals were grown by solution evaporation. Equimolar (0.300 mmol) amounts of FS and PA were refluxed in acetone at approximately 80 °C for 15 minutes. Solutions were then left to evaporate at room temperature. The same method was repeated with ethanol. In each case, clear, rectangular, plate-like crystals appeared after 48 hours. Single crystal X-ray diffraction data on FSPA acetone were collected using an Oxford Diffraction Xcalibur Gemini Diffractometer, with Mo radiation, while those on FSPA ethanol were collected on beamline I19 at Diamond Light Source, using a Rigaku Saturn 724+ diffractometer and wavelength 0.68890 Å. In each case, samples were cooled to 120 K using an Oxford Cryosystems Cryostream 600 device.

**Table S1** Key crystallographic information for FSPA solvates structure refinements

| | FSPA acetone | FSPA ethanol |
|---|---|---|
| Empirical formula[a] | $C_{18}H_{17}N_4O_6SCl$ | |
| Molar mass[a] | 452.9 g mol$^{-1}$ | |
| Temperature | 120 K | |
| Crystal system | Triclinic | |
| Space group | $P\bar{1}$ | |
| *a* / Å | 5.0474(17) | 5.2086(17) |
| *b* / Å | 14.428(2) | 14.641(5) |
| *c* / Å | 14.778(3) | 14.656(5) |
| α / ˚ | 76.344(14) | 76.378(5) |
| β / ˚ | 83.49(2) | 87.592(3) |

| | | |
|---|---|---|
| γ / ° | 86.854(19) | 83.243(3) |
| Volume / Å$^3$ | 1038.5(4) | 1078.6(6) |
| *Z* | 2 | 2 |
| Reflections collected | 6807 | 3660 |
| Observed reflections | 2585 | 3197 |
| Parameters | 268 | 267 |
| Goodness of fit | 1.03 | 1.08 |
| *R* indices (*I*/σ = 2) | *R* = 0.1298<br>$R_w$ = 0.1485 | *R* = 0.1011<br>$R_w$ = 0.1052 |

[a] Solvent molecules not included.

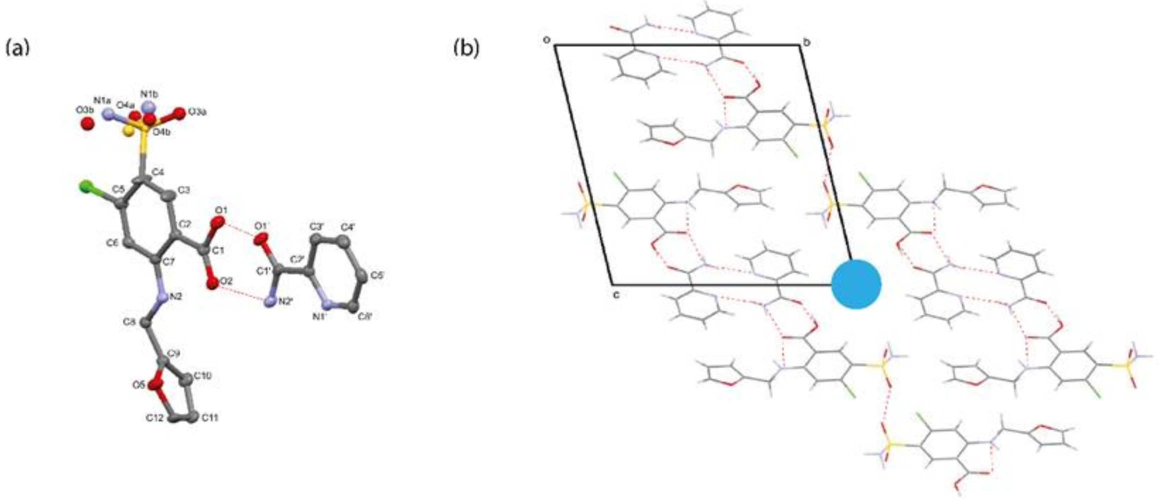


**Figure S1** (a) Asymmetric unit of the FSPA solvates, with thermal ellipsoids shown at the 50% probability level. Disorder of the sulfonamide group is shown with split-atom sites (only one orientation is shown for clarity), and hydrogen bonds are indicated by red dashed lines. (b) Packing diagram viewed along the *a* axis. The cyan circle indicates the centre of the unmodelled electron density corresponding to the solvent.

As shown in Fig. S1, the shortest hydrogen bonding interaction is between the carboxylic acid of FS and the amide of PA (O1H1···O1′), with $d_{O\cdots O}$ = 2.55(1) Å in the acetone solvate and $d_{O\cdots O}$ = 2.592(5) Å in the ethanol solvate. Rotation about the FS C1–C2 bond is constrained by the presence of an intramolecular hydrogen bond (N2H···O2, $d_{N\cdots O}$ = 2.726(9) Å and $d_{N\cdots O}$ = 2.767(5) Å in the acetone and ethanol solvates respectively).

The system forms ribbons of alternating pairs of FS molecules connected to pairs of PA molecules. Neighbouring FS molecules form close contacts between sulfonamide groups (O3···O3, $d_{O\cdots O}$ = 2.16(2) Å) and neighbouring PA molecules interact in a dimer formation (N2′H···N1′, $d_{N\cdots N}$ = 3.10(1) Å). The ribbons are loosely connected through the sulfonamide groups *via* N1H···O3 ($d_{N\cdots O}$ = 2.68(3) Å) hydrogen bonds. The bond distances given here are for the acetone solvate, but the same hydrogen bonding is present in the ethanol solvate, with acceptor-donor distances changing by less than 2%. The N1H···O3 interaction is the exception with $d_{N\cdots O}$ increasing by 12% in the ethanol solvate, reflecting a small elongation in the *a* axis, seen in Table 1. The sulfonamide group is refined with disorder over two positions with (fixed) equal occupancies, similar to the disorder observed in the furosemide.isonicotinamide cocrystal[3,4] but with a larger angle between the two orientations (110° instead of 32°).

Note that a very similar structure has been reported by Banik et al. for FSPA sesquihydrate[20].

## 3. $^{13}$C NMR of FSPA solvates

Evidence of cocrystal formation is seen in Fig. S2, since the FSPA acetone and ethanol solvates show distinct $^{13}$C SSNMR spectra compared to the starting materials and the parent FSPA cocrystal. The peaks of pure PA are weak due to its extremely long $^1$H $T_1$ relaxation (of the order of 100 s). The solvate

spectra are broad compared to FSPA and pure FS (linewidths of 150–200 Hz), but no peak splitting is observed at the carbonyl region (C1, 172.2 ppm and C1', 169.9 ppm). This is in keeping with the asymmetric unit containing a single FS and a single PA molecule. The coloured arrows indicate additional peaks that correspond to the solvent molecules. Assignment details can be found in Ref. [5]. The acetone carbonyl peak is not observed but should appear at around 200 ppm; previous literature of acetone solvates has also shown weak acetone carbonyl signals, ascribed to inefficient cross-polarisation (CP) due to motional processes interfering with the magnetisation transfer during the contact time.[6] Little difference is observed in the FS and PA chemical shifts of the two solvates, in keeping with the isostructural relationship observed from SCXRD data.

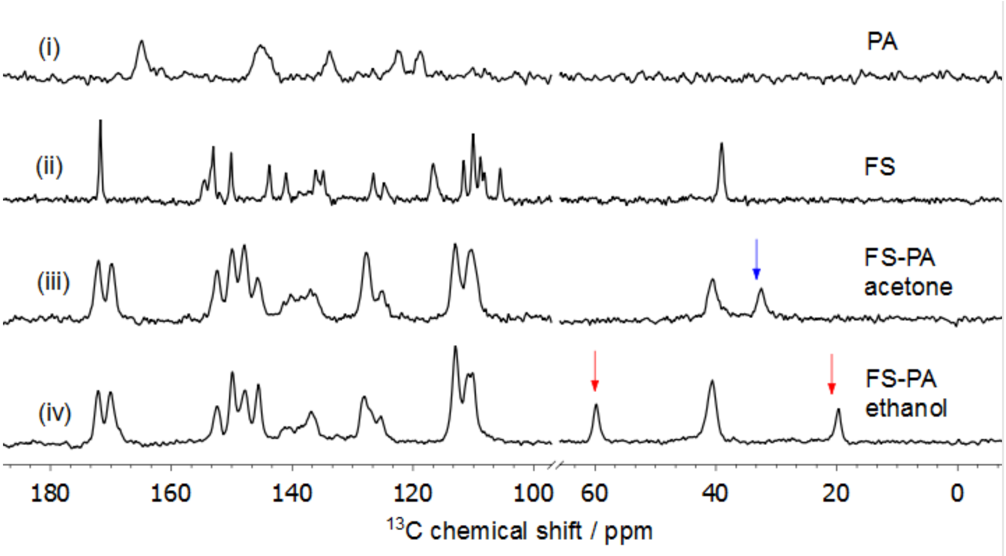


**Figure S2** $^{13}C$ CP/MAS spectra of (i) PA, (ii) FS, (iii) FSPA acetone solvate and (iv) FSPA ethanol solvate acquired at 125.7 MHz and ambient probe temperature. Spectra (i)–(iii) were acquired at 8 kHz MAS and spectrum (iv) at 5 kHz MAS. The recycle delay was 5 s and contact time 2.5 ms over 100 transients for all except (i) recycle delay 30 s with contact time 4 ms, (ii) recycle delay 15 s with contact time 4 ms and (iv) 480 transients. The blue arrow indicates the acetone $CH_3$ peak and the red arrows indicate the ethanol peaks.

Quantitative $^{13}C$ MAS NMR experiments showed that the fraction of solvent voids occupied by solvent molecules was about 80% in samples left to dry for a few weeks (measurements of solvent fraction in very fresh samples were distorted by adventitious solvent). The solvent fraction slowly decayed over a timescale of weeks, implying that the solvates are non-stoichiometric and stable down to relatively low occupancies. Over time the solvent peak intensities decreased, with no changes observed to the rest of the spectrum, which is consistent with a non-stoichiometric solvate. No degradation was observed via $^{13}C$ SSNMR over a 9-month period, but the solvates are, however, unstable above 80 °C, degrading to a mixture of materials, including an unsolvated FSPA co-crystal and pure FS. These NMR and thermal studies are described in detail in Ref. [5].

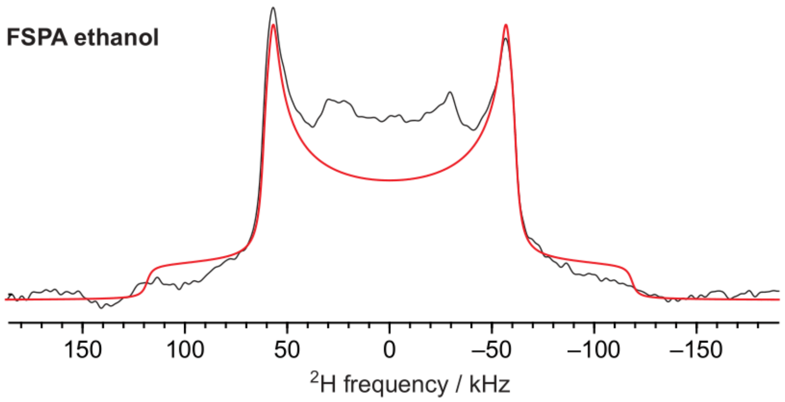


**Figure S3** Low temperature (185 K) FSPA-ethanol spectrum fitted to $C_Q$ = 158 kHz, $\eta$= 0.04. Note that the feature in the centre of the spectrum is due to dynamic solvent molecules, as discussed in the main text. The asymmetry parameter is negligibly small, and so η = 0 was used in calculations.

## 4. Fitting of kinetic parameters from $^2$H relaxation data

The well-defined "$T_1$ minimum" of Fig. 4(a) (main text) is consistent with a thermally activated process with a single correlation time, $\tau_c$, whose temperature dependence can be described by an Arrhenius-like equation, $\tau_c = \tau_\infty \exp(E_a/RT)$. The $T_1$ values were calculated with the standard expression:

$$\frac{1}{T_1} = A[J(\nu_0) + 4J(2\nu_0)]$$

where the leading terms in the corresponding equation in the main text, Eq. (4), are combined into the scaling / amplitude factor $A$, and $J(\nu)$, the spectral density, is defined (in frequency units) as

$$J(\nu) = \frac{2\tau_c}{1 + (2\pi\nu\tau_c)^2}$$

This is equivalent to Eqs. (5)–(7) of the main text for isotropic rotational diffusion, i.e. exponential decay of $C_2(t)$ to zero ($S^2$ = 0).

The temperature dependence of the relaxation times is commonly fitted directly to the parameters $\tau_\infty$ (often denoted $\tau_0$) and $E_a$ (the activation energy), although the stability of the fitting is improved if the fitting parameter is log $\tau_\infty$. This parameterisation has a few significant drawbacks. Firstly, $\tau_\infty$ (the notional correlation time at infinite temperature) is not a physical significant parameter, and so suitable initial values are generally chosen by trial and error. Secondly, it is very strongly correlated with the $E_a$ parameter, which compromises the numerical stability of the fitting and further complicates the identification of suitable starting values. This correlation also makes it difficult to determine uncertainties on values calculated from the fitted parameters, such as correlation times and jump rates; classical "error propagation" formulae are only valid for independent, uncorrelated variables.

Hence, we have parameterized the relaxation fittings using a parameter $T_{min}$, the temperature at which the correlation time is the reciprocal of the (angular) Larmor frequency,

$$\tau_c(T_{min}) = \frac{1}{2\pi\nu_0}$$

This will approximately correspond to the temperature at which $T_1$ has its minimum, allowing the initial estimate of $T_{min}$ to be determined directly from the data. The temperature dependence of $\tau_c$ in this formulation is then

$$\tau_c = \tau_c(T_{min})\exp\left(\frac{E_a}{R}\left(\frac{1}{T} - \frac{1}{T_{min}}\right)\right)$$

Since the activation energy is determined by the gradient of the curve and $T_{min}$ is determined by the location of the minimum in the horizontal axis, the correlation between the parameters is significantly reduced.

Figure S4 shows a Monte Carlo analysis of the fitting of the data in Fig. 4 (a) of the main text. There is a major reduction in correlation using the revised fitting parameters; $E_a$ and $\log_{10}(\tau_\infty)$ (left) are very strongly anti-correlated, while the correlation between $E_a$ and $T_{min}$ is minimal. As shown in Table S2, the re-parameterisation does not affect the values of the fitted parameters or their uncertainties.

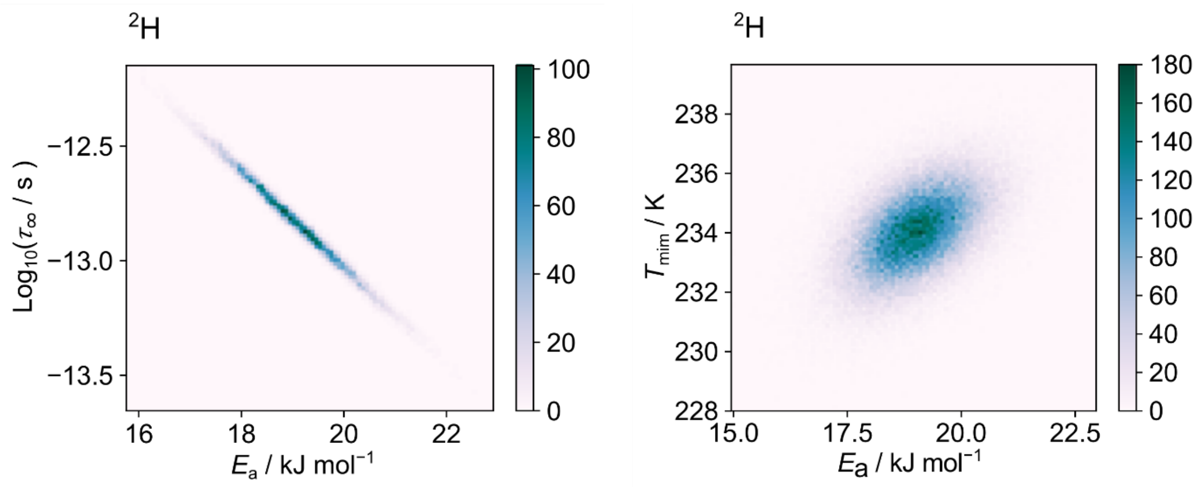


**Figure S4** Monte Carlo simulation of the effect of random noise on the fitted parameters using synthetic data corresponding to the $T_1$ relaxation data of Fig. 4(a) of the main text. (Left) using the conventional parameters $E_a$ and $\log_{10}(\tau_\infty)$ (10 000 simulations) and (right) fitting $E_a$ and $T_{min}$ (100 000 simulations). The magnitude of random noise added was based on the residuals from the fit of the experimental data. The $A$ parameter is not strongly correlated with the other parameters and is not considered. The vertical probability density scale is arbitrary.

**Table S2** Results from fitting the data of Fig. 4 (a) using the alternative parameterisations. Uncertainties are given to one standard error.

| $\log_{10}(A$ / Hz$^2)$ | $E_a$ / kJ mol$^{-1}$ | | Correlation coefficient between $E_a$ and $\log_{10}(\tau_\infty)$ or $T_{min}$ |
|---|---|---|---|
| 9.657 ± 0.014 | 19.0 ± 0.9 | $\log_{10}(\tau_\infty$ / s) = –12.82 ± 0.19 | –0.9947 |
| 9.657 ± 0.014 | 19.0 ± 0.9 | $T_{min}$ = 234.0 ± 1.2 K | 0.49 |

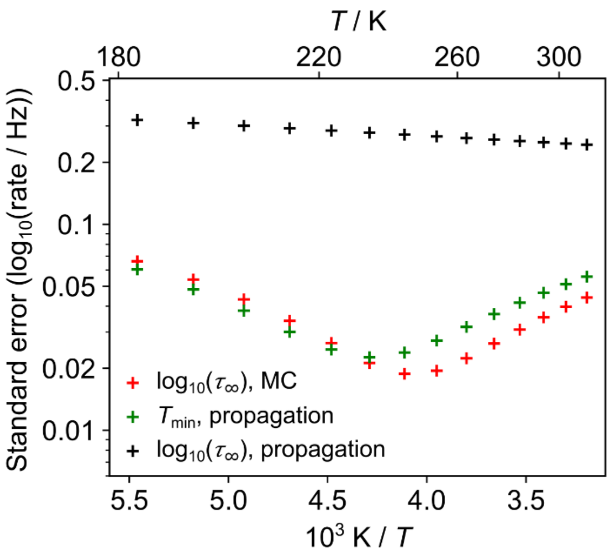


**Figure S5** Variation of the standard error in the predicted rate of solvent motion, $\nu = 1/(2\pi\tau_c)$, in FSPA ethanol-$d_2$ with temperature based on the fitting of Fig. 4(a). Uncertainty parameterisation using the $T_{min}$ parameterisation (green makers) gives results that closely match the Monte Carlo simulations (red markers). Naïve uncertainty propagation based on $\tau_\infty$ (black markers) gives incorrect results. Note that MC analysis gives identical results independently of the parameters used (within the convergence limits of MC simulation).

The "rate" of isotropic motion in terms of the fitted parameters is given by:

$$\nu = \frac{\nu_0}{\exp\left(\frac{E_a}{R}\left(\frac{1}{T} - \frac{1}{T_{min}}\right)\right)}$$

Classic uncertainty propagation, which assumes that $E_a$ and $T_{min}$ are uncorrelated gives the uncertainty on $\log_{10}(\nu)$ as:

$$\delta \log_{10} \nu = \frac{1}{RT \ln 10}\sqrt{\left(\delta E_a(T - T_{min})\right)^2 + \left(\frac{\delta T_{min} E_a T}{T_{min}}\right)^2}$$

Figure S5 compares the predictions of this equation with the results of a straightforward, if much slower, Monte Carlo (MC) approach to estimating the uncertainty in $\log_{10}(\nu)$, on the assumption that random errors are dominant. The limited correlation between the parameters means that there is good overall agreement, and the uncertainty estimates can be determined without requiring MC simulations. Naïve use of error propagation with the normal $\tau_\infty$ formulation gives very different, and erroneous, results. Note how the random noise has least effect on calculated rates around the relaxation minimum, but its effect increases monotonically and significantly (noting the logarithmic scale) with distance from the minimum.

## 5. $^2$H NMR data and simulations

### Prediction of NMR spectra from MD simulations

This trajectory-based approach for prediction of the spectral lineshapes employs the Liouville von Neumann equation in the semi-classical approximation, often called the Langevin form of the Stochastic Liouville Equation:[7,8]

$$\frac{d\boldsymbol{\rho}(t)}{dt} = -i\hat{\hat{L}}(t)\boldsymbol{\rho}(t) \tag{S1}$$

where $\boldsymbol{\rho}(t)$ is a density matrix of the system and the Liouvillian $\hat{\hat{L}}$ is a superoperator of the interaction Hamiltonian (expressed in units of $\hbar$).

$^2$H NMR probes dynamics via the coupling between electric field gradient tensor, **V**, at the given site and the nuclear electric quadrupolar moment. Ignoring the effects of longitudinal relaxation, the only non-zero matrix elements of $\hat{\hat{L}}$ relevant for the time evolution of the transverse magnetisation involve:

$$\nu_{\pm 1}(t) = \pm\frac{1}{2}\chi\big((3\cos^2\beta_{\mathrm{P}\to\mathrm{L}}(t) - 1) - \eta\sin^2\beta_{\mathrm{P}\to\mathrm{L}}(t)\cos 2\gamma_{\mathrm{P}\to\mathrm{L}}(t)\big) \quad \text{(S2)}$$

which are the frequencies of the two allowed NMR transitions for *I* = 1. The Euler angles $\Omega_{\mathrm{P}\to\mathrm{L}}(t)$ define the passive rotation from the interaction PAS frame to the laboratory-fixed frame for each time point on the MD trajectory. $\chi$ and $\eta$ are the conventional parameters defining the magnitude of the EFG tensor **V**:

$$\chi = \frac{eV_{ZZ}^{\mathrm{P}}Q}{h} \qquad \eta = \frac{V_{XX}^{\mathrm{P}} - V_{YY}^{\mathrm{P}}}{V_{ZZ}^{\mathrm{P}}} \quad \text{(S3)}$$

where the right-handed axis set, *X*, *Y*, *Z*, aligned with principal components, has been ordered with the convention $|V_{ZZ}^{\mathrm{P}}| \geq |V_{XX}^{\mathrm{P}}| \geq |V_{YY}^{\mathrm{P}}|$ (this choice of ordering is not physically significant).

As shown by Oganesyan[7,8], the evolution of the spin density matrix in the fast motional limit can be calculated using the expression:

$$\boldsymbol{\rho}(t) = \exp\left(-\left[i\,\langle\hat{\hat{L}}\rangle + \hat{\hat{\Lambda}}\right]t\right)\boldsymbol{\rho}(0) \quad \text{(S4)}$$

The first term in the matrix exponential, $\langle\hat{\hat{L}}\rangle = \frac{1}{\tilde{T}}\int_0^{\tilde{T}}\langle\hat{\hat{L}}(\tau)\rangle\,d\tau$, is a Liouvillian which is averaged over the time of complete relaxation of the correlation function of the molecular motion ($\tilde{T} \approx 10\tau_c$) and over the *N* copies of the molecules in the simulation; this describes the "average" evolution. This first term is equivalent to deriving averaged NMR parameters from molecular trajectories, which is sufficient if the correlation times are extremely short. In the context of $^2$H NMR, however, it is important to describe the line-broadening effects of the dynamics, which is captured by the "decoherence matrix" $\hat{\hat{\Lambda}} = \int_0^{\tilde{T}}\langle\Delta\hat{\hat{L}}(0)\Delta\hat{\hat{L}}(\tau)\rangle\,d\tau$ of the second term. $\Delta\hat{\hat{L}}(\tau) = \hat{\hat{L}}(\tau) - \langle\hat{\hat{L}}\rangle$ defines the dephasing of the magnetisation caused by the modulation of $\hat{\hat{L}}(\tau)$ due to the re-orientational dynamics of the molecule.

The density matrix is propagated according to Eq. (S4) using standard numerical approaches, and projected onto the detection operator $\hat{I}_+$ , in accordance with $\langle I_+\rangle = Tr\big(\hat{\boldsymbol{I}}_+\boldsymbol{\rho}\big) = \rho_{0,+1} + \rho_{0,-1}$, to generate the NMR signal, *s*(*t*), prior to Fourier transformation into the frequency domain. In this simple case of only non-zero secular terms $\nu_{\pm 1}(t)$, however, the $\langle\hat{\hat{L}}\rangle$ and $\hat{\hat{\Lambda}}$ matrices are diagonal, with the $\bar{\nu}_{\pm 1}$ corresponding to the averaged NMR frequencies with associated damping coefficients $\lambda_+ = \lambda_- = \lambda$ calculated from:

$$\lambda = \int_0^{\tilde{T}}\langle\Delta\nu_{\pm 1}(0)\Delta\nu_{\pm 1}(\tau)\rangle\,d\tau \quad \text{(S5)}$$

where $\Delta\nu_{\pm 1}(t) = \nu_{\pm 1}(t) - \bar{\nu}_{\pm 1}$ . The resulting expressions for the required density matrix elements are as follows:

$$\rho_{0,\pm 1}(t) = \exp(-[i\bar{\nu}_{\pm 1} + \lambda]t)\rho_{0,\pm 1}(0) \quad \text{(S6)}$$

The following procedure is used to calculate the NMR response for a given MD trajectory. Trajectories from all individual molecules are first combined into a single continuous one by performing appropriate rotational transformations. Then the quadrupolar coupling tensor is averaged according to the following equation[9]:

$$\left(\mathbf{V}^{\mathrm{A}}\right)_{ij} = V_{XX}^{\mathrm{P}}\,\langle l_{xi} l_{xj}\rangle + V_{YY}^{\mathrm{P}}\,\langle l_{yi} l_{yj}\rangle + V_{ZZ}^{P}\,\langle l_{zi} l_{zj}\rangle \tag{S7}$$

i.e., time averages are performed on the products of the projection cosines, $l_{ij}$, of the three PAS axes in the lab frame. The averages of the time-dependent frequencies $\bar{v}_{\pm 1}$ and the dumping coefficients $\lambda_{\pm}$ are then calculated using the principal values and their axes of the averaged tensor, $\mathbf{V}^{\mathrm{A}}$. In particular, the averages of the time-dependent frequencies of Eq. (S2) can be written:

$$\bar{v}_{\pm 1}(\Omega_{\mathrm{A}\to\mathrm{L}}) = \pm\frac{1}{2}\langle\chi\rangle\big((3\cos^2\beta_{\mathrm{A}\to\mathrm{L}} - 1) - \langle\eta\rangle\sin^2\beta_{\mathrm{A}\to\mathrm{L}}\cos 2\gamma_{\mathrm{A}\to\mathrm{L}}\big) \tag{S8}$$

where $\langle\chi\rangle$ and $\langle\eta\rangle$ are calculated from the principal values of $\mathbf{V}^{\mathrm{A}}$ (cf. Eq. S3), and $\beta_{\mathrm{A}\to\mathrm{L}}$ and $\gamma_{\mathrm{A}\to\mathrm{L}}$ are Euler angles defining the orientation of the laboratory *z* axis in the frame of the principal axes of $\mathbf{V}^{\mathrm{A}}$; this corresponds to the orientation of the crystallite with respect to the lab frame.

The damping coefficients (non-zero elements of the decoherence matrix) are calculated according to Eq. (S5) where now $\Delta\nu_{\pm 1}(t) = \nu_{\pm 1}(\Omega_{\mathrm{P}\to\mathrm{A}}(t);\Omega_{\mathrm{A}\to\mathrm{L}}) - \bar{v}_{\pm 1}(\Omega_{\mathrm{A}\to\mathrm{L}})$ and the time averaging is performed over rotations in the A frame which are defined by $\Omega_{\mathrm{P}\to\mathrm{A}}(t)$. The total rotational transformation from the PAS to the lab frame is defined as $R(\Omega_{\mathrm{P}\to\mathrm{L}}(t)) = R(\Omega_{\mathrm{A}\to\mathrm{L}})R(\Omega_{\mathrm{P}\to\mathrm{A}}(t))$. Note that both parameters $\bar{v}_{\pm 1}(\Omega_{\mathrm{A}\to\mathrm{L}})$ and $\lambda_{\pm}\,(\Omega_{\mathrm{A}\to\mathrm{L}})$ are functions of $\beta_{\mathrm{A}\to\mathrm{L}}$ and $\gamma_{\mathrm{A}\to\mathrm{L}}$ .

Finally, the response is averaged over $\beta_{\mathrm{A}\to\mathrm{L}}$ and $\gamma_{\mathrm{A}\to\mathrm{L}}$ angles assuming an isotropic distribution of crystallites in the sample.

These $^2$H NMR simulations are an integrated feature of the MD-EPR simulation software suite SpinMolDyn[10].

**Prediction of $T_1$ relaxation times from MD simulations**

For the prediction of $T_1$ relaxation times from the results of MD simulations, we have adapted the Model-free approach developed by Lipari and Szabo for the interpretation of NMR relaxation[11]. In the case of restricted motion of a molecule in a liquid crystalline solution with overall isotropic rotation the total correlation function can be presented as:

$$C_{\mathrm{T}}(t) = \frac{1}{5}\big(S^2 + (1 - S^2)C(t)\big)e^{-t/\tau_{\mathrm{G}}} \tag{S9}$$

where $S^2$ is the square of the generalised order parameter of the molecular orientations, $\tau_{\mathrm{G}}$ is the correlation time of the overall isotropic rotation and $C(t) = \big(C_2(t) - C_2(\infty)\big)/\big(C_2(0) - C_2(\infty)\big)$ is the normalised re-orientational correlation function of internal motions. Note that for an isotropic rigid distribution of crystallites in the sample, as in this work, $\tau_{\mathrm{G}} \to \infty$ , $e^{-t/\tau_{\mathrm{G}}} \to 1$ and $C_{\mathrm{T}}(t) \to C_2(t)/5$.

$C_2(t)$ is defined as:

$$C_2(t) = \lim_{T\to\infty}\frac{1}{T}\int_0^T \langle P_2\big(\vec{\mu}(\tau)\cdot\vec{\mu}(t+\tau)\big)\rangle d\tau \tag{S10}$$

$P_2(t)$ is a second-order Legendre polynomial and the average is taken over MD simulation time, using a 'sliding time window', and the number of molecules in the system.

Within the framework of Redfield approximation, the relaxation times are calculated using the spectral densities of the correlation function which are the one-sided Fourier transforms of $C_{\mathrm{T}}(t)$:

$$J(\omega) = 2\int_0^\infty C_{\mathrm{T}}(t)\cos(\omega t)\,dt \tag{S11}$$

The spectral density function of the first term in $C_{\mathrm{T}}(t)$ in Eq. (S9) is:

$$\frac{2}{5}\left(\frac{S^2\tau_\mathrm{G}}{1+(\omega_0\tau_\mathrm{G})^2}\right) \tag{S12}$$

This term tends to zero for sufficiently large $\tau_\mathrm{G}$ and, therefore, can be ignored in the calculation of $T_1$ for an isotropic distribution of crystallites in the sample. Note that this approach is valid for any value of $\tau_\mathrm{G}$ as long as the conditions of the Redfield approximation for $T_1$ are satisfied, namely, the total effective correlation time (which is $\approx \tau$, the effective correlation time of the fast internal motional, when $\tau_\mathrm{G} \gg \tau$) is within the fast motional limit[11]; $2\pi|\langle\Delta\nu_{\pm1}(0)\rangle|\tau \ll 1$ and $2\pi|\langle\Delta\nu_{\pm1}(0)\rangle| \ll \omega_0$.

The longitudinal relaxation times $T_1$ of $^2$H due to quadrupolar interactions under the Redfield approximation and in the case of restricted local molecular motions are given by the standard expression[12,13]:

$$\frac{1}{T_1}=\frac{3\pi^2}{20}\chi^2(1-S^2)\left(1+\frac{\eta^2}{3}\right)(J(\omega_0)+4J(2\omega_0)) \tag{S13}$$

where $\omega_0$ is the NMR frequency (expressed as an angular frequency) and $J(\omega)$ is the spectral density function of $C(t)$. The one-sided Fourier transformation of the correlation function $C(t)$, which combines multiple contributions to the rotational motion of $\vec{\mu}(t)$, is carried out numerically.

**Prediction of NMR spectra from simple jump-based Markov models**

Initial analysis used EXPRESS[14] software to model the lineshapes in terms of stochastic jumps between a limited number of Markov states. As discussed in the main text this approach to calculating the lineshape is quite different from the "direct prediction" approach. Similarly, it differs from the "Markov State Modelling" analysis using pyEMMA, which derives Markov models from the MD trajectory, rather than being postulated.

The parameters used to simulate the $^2$H spectra of the acetone solvate by modelling a $C_2$ jump about the C=O axis are shown in Table S3. Figure S6 shows the result of adjusting the apparent jump rate of this process to match the experimental spectra, illustrating how a model based on intermediate timescale dynamics seems to describe motion which, in retrospect, is on a fast (ns) timescale. Note that the $C_\mathrm{Q}$ value in Table S3 was estimated from a DFT calculation (186 kHz) scaled by the methyl $C_3$ rotation. This is probably an over-estimate compared to the conventional literature value of 160 kHz for the $C_\mathrm{Q}$ of $^2$H in a methyl group used in the main text, but will have negligible impact on the qualitative (and, as argued, erroneous) matching of simulated and experimental spectra.

**Table S3** Parameters for EXPRESS simulation of a simple two-site jump model of motion in FSPA acetone solvate.

| | | | |
|---|---|---|---|
| Experiment | $^2$H quadrupole echo | | |
| $\tau_{90^\circ}$ / µs | 3 | | |
| Echo delay / µs | 60 | | |
| Dwell time / µs | 2.5 | | |
| $\nu_{2H}$ / MHz | 76.71 | | |
| Recycle delay / s | 1 | | |
| Euler angles/ ° | α | β | γ |
| Site 1 | 0 | 0 | 0 |
| Site 2 | 0 | 115 | 0 |
| Relative weighting | 0.5 | | |
| $C_Q$ / kHz | 62 | | |
| η | 0 | | |
| Power sampling scheme | ZCW 500 | | |
| Left shift | 24 | | |

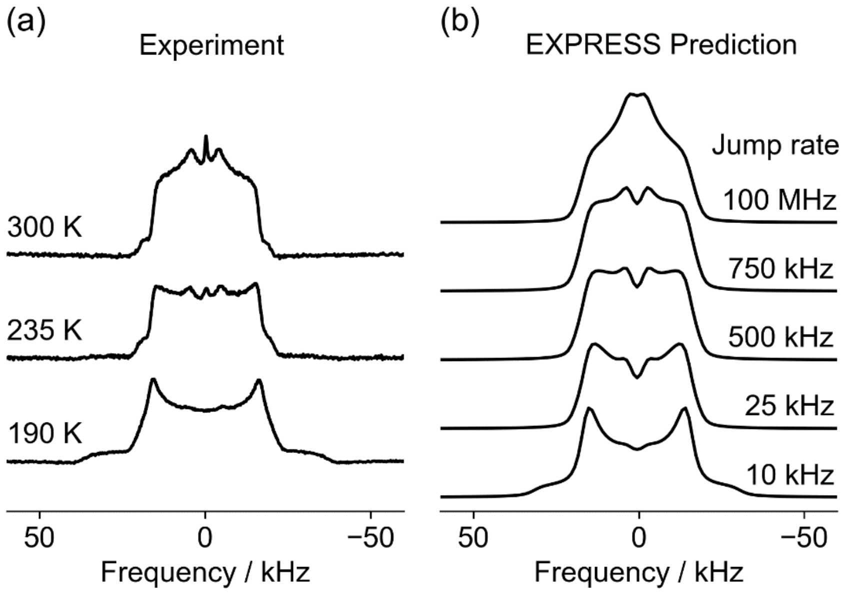


**Figure S6.** (a) Variable temperature static $^2$H spectra of FSPA acetone-$d_6$ solvate acquired with an echo delay τ = 60 µs over 72000 transients. (b) EXPRESS-simulated static $^2$H spectra over a two-site jump motion of 115° using the parameters in Table S3. The jump rates given provided good visual matches to the experimental spectra. As discussed in the text, however, these rates are *not* physically meaningful, since effectively the wrong equation is being applied to generate the spectra. Figure adapted from Ref. 3.

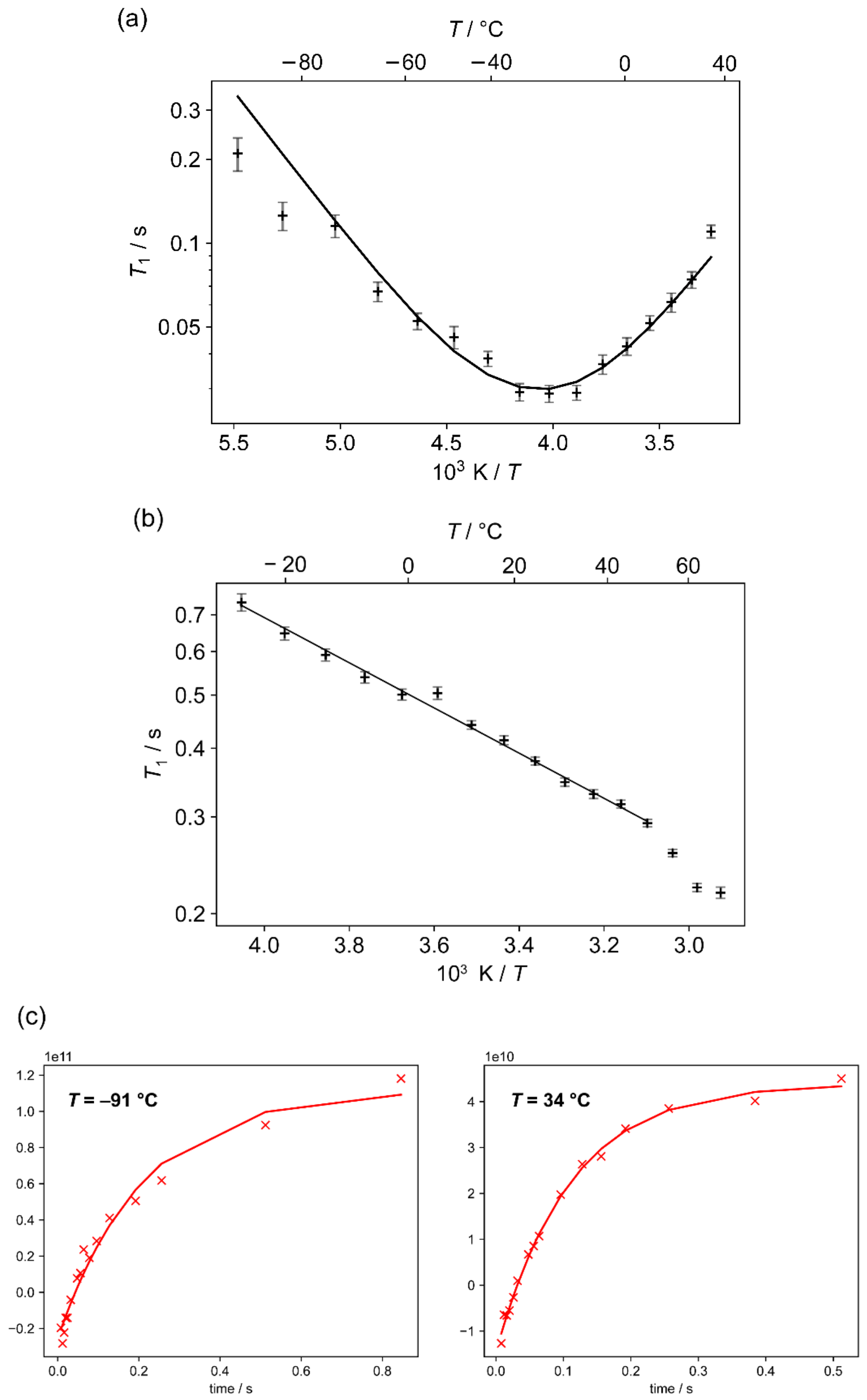


**Figure S7** $^{2}H$ spin-lattice relaxation time constants as a function of temperature of (a) FSPA ethanol-*d*$_2$ at 61.42 MHz as a function of temperature. The curve shows a fit to $E_a$ of 19.0 ± 0.9 kJ mol$^{-1}$ and a $T_{min}$ = 234.0 ± 1.2 K. As noted in the main text, there are increasing contributions from "frozen" solvent molecules in the low temperature half of the $T_1$ minimum. Although the $E_a$ value is likely to be reasonably well defined by the high temperature half, the $T_{min}$ is more suspect, and is in clear disagreement with the MD predictions. (b) Analogous data for FSPA acetone-*d*$_6$ acquired at 76.8 MHz. The straight line is a fit to $E_a$ of 7.9 ± 0.2 kJ mol$^{-1}$. (c) Sample fits for FSPA ethanol-*d*$_2$ showing biexponential character at low temperature data.

## Principal values of the rotationally and flip-averaged quadrupolar coupling tensors

The expressions below give the motionally averaged components of the quadrupolar tensor as a function of the Euler angles relating the static and rotating axis systems[15].

**Free stochastic rotation about a single axis:**

The principal values of the rotationally averaged tensor along a single axis in which $\alpha$ and $\beta$ are the Euler angles relating the components of the **V** tensor in its molecule-fixed / PAS frame, $\mathbf{V}^{\mathrm{P}}$, (axes *X*, *Y* and *Z*) and the motionally averaged $\mathbf{V}^{\mathrm{R}}$ tensors (axes *x*, *y* and *z*) respectively[15,16].

$$V_{xx}^{\mathrm{R}} = V_{yy}^{\mathrm{R}} = \frac{1}{2}\left(V_{XX}^{\mathrm{P}}(1 - \cos^2\alpha \sin^2\beta) + V_{YY}^{\mathrm{P}}(1 - \sin^2\alpha \sin^2\beta) - V_{ZZ}^{\mathrm{P}}\sin^2\beta\right) \qquad \text{(S14)}$$

$$V_{zz}^{\mathrm{R}} = V_{XX}^{\mathrm{P}}\cos^2\alpha \sin^2\beta + V_{YY}^{\mathrm{P}}\sin^2\alpha \sin^2\beta + V_{ZZ}^{\mathrm{P}}\cos^2\beta$$

**Flips between two equivalent sites:**

The principal values of the flip-averaged tensor, where $\alpha$ is the angle between the molecule-fixed / PAS frame and the motionally averaged $V_{ZZ}^{\mathrm{P}}$ and $V_{zz}^{\mathrm{F}}$ components of the tensors, respectively[15,16]:

$$V_{xx}^{\mathrm{F}} = V_{XX}^{\mathrm{P}}\cos^2\left(\frac{\alpha}{2}\right) + V_{ZZ}^{\mathrm{P}}\sin^2\left(\frac{\alpha}{2}\right) \qquad \text{(S15)}$$

$$V_{yy}^{\mathrm{F}} = V_{YY}^{\mathrm{P}}$$

$$V_{zz}^{\mathrm{F}} = V_{XX}^{\mathrm{P}}\sin^2\left(\frac{\alpha}{2}\right) + V_{ZZ}^{\mathrm{P}}\cos^2\left(\frac{\alpha}{2}\right)$$

## Detailed results from direct simulation methodology

As expected, the principal values of the motionally averaged tensor, $\mathbf{V}^{\mathrm{P}}$, steadily decrease with temperature due to increased motional averaging. This is reflected in the steadily narrowing lineshapes of Figs. S8 and S9, and more directly in the tabulated values in Table S4. It is not possible, however, to rationalise these values in terms of simple motional models, such as free rotational diffusion about an axis, or two-fold jumps around molecule-fixed axes, such as the bisector of the H–C–H plane in ethanol or the $C_2$ symmetry axis of acetone. Calculated averaged principal components for such motions, Table S5, show that rotational diffusion gives values that are too small. In the case of acetone, two-fold jumps about the symmetry axis gives principal values (3.52, 19.96 and 23.48 kHz) that are comparable with values obtained in the 300 K simulation (averages over A and B models are 5.8, 12.6 and 18.5 kHz). This is consistent with the two-state Markov model simulations for this system at 300 K (cf. Fig. S6 above). This is, however, the only point of contact between simple motional models and results of MD simulation.

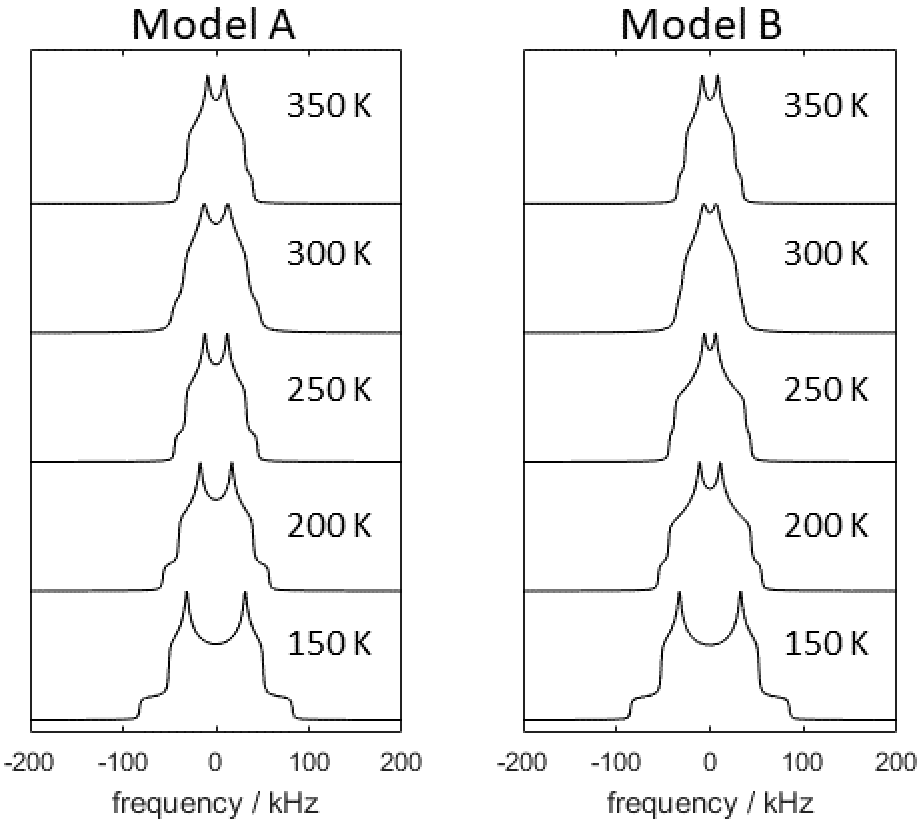


**Figure S8**. $^{2}H$ quadrupolar NMR spectral lineshapes for FSPA ethanol-$d_2$ predicted for model A (left panel) and model B (right panel).

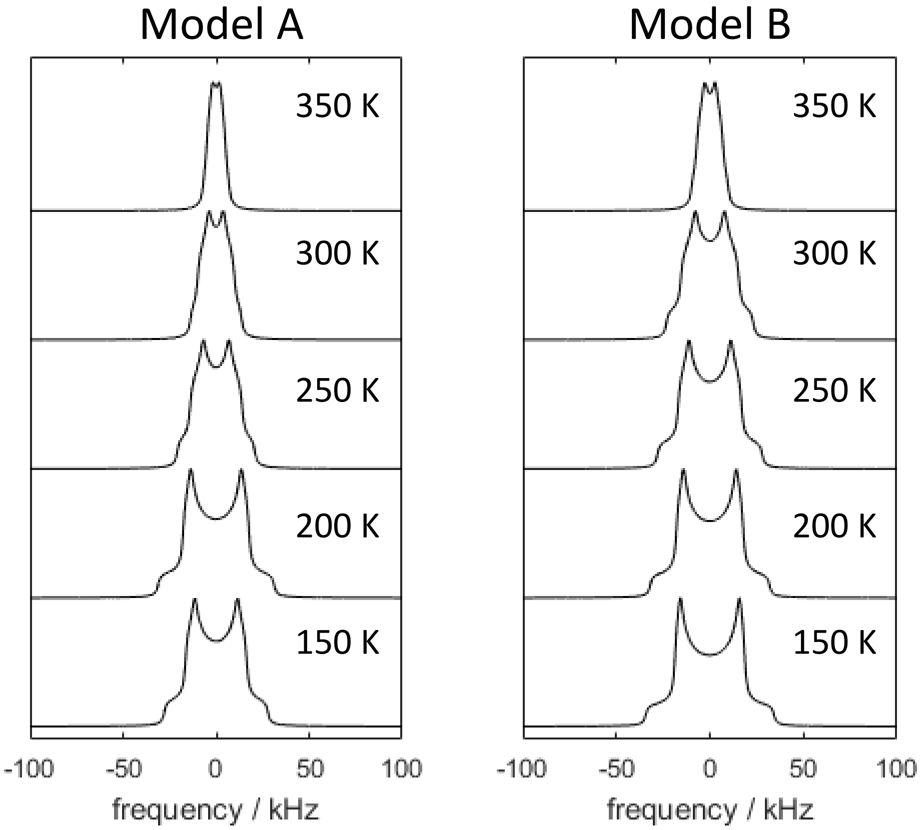

**Figure S9**. $^{2}H$ quadrupolar NMR spectral lineshapes for FSPA acetone-$d_6$ predicted for model A (left panel) and model B (right panel).

**Table S4.** Magnitudes of principal components of the motionally averaged $\mathbf{V}^{\mathrm{A}}$ tensor in kHz (given in increasing order).

| | 150 K | 200 K | 250 K | 300 K | 350 K |
|---|---|---|---|---|---|
| ETH –<br>Model A | 31.3875<br>50.7478<br>82.1348 | 16.9914<br>40.0830<br>57.0732 | 12.0809<br>32.5453<br>44.6268 | 12.8441<br>34.3046<br>47.1496 | 8.9724<br>30.8847<br>39.8571 |
| ETH –<br>Model B | 32.6909<br>52.2771<br>84.9673 | 11.1365<br>44.0987<br>55.2350 | 6.1270<br>37.7126<br>43.8398 | 7.0061<br>29.0169<br>36.0240 | 8.3058<br>26.3166<br>34.6228 |
| ACE –<br>Model A | 11.4312<br>16.4208<br>27.8523 | 13.5490<br>17.6471<br>31.1963 | 6.8415<br>13.9712<br>20.8121 | 3.8515<br>9.8399<br>13.6912 | 1.9183<br>4.8991<br>6.8179 |
| ACE –<br>Model B | 15.7757<br>18.5553<br>34.3312 | 14.0986<br>17.8170<br>31.9148 | 11.1724<br>16.8667<br>28.0394 | 7.7992<br>15.4633<br>23.2623 | 2.8354<br>6.9692<br>9.8042 |

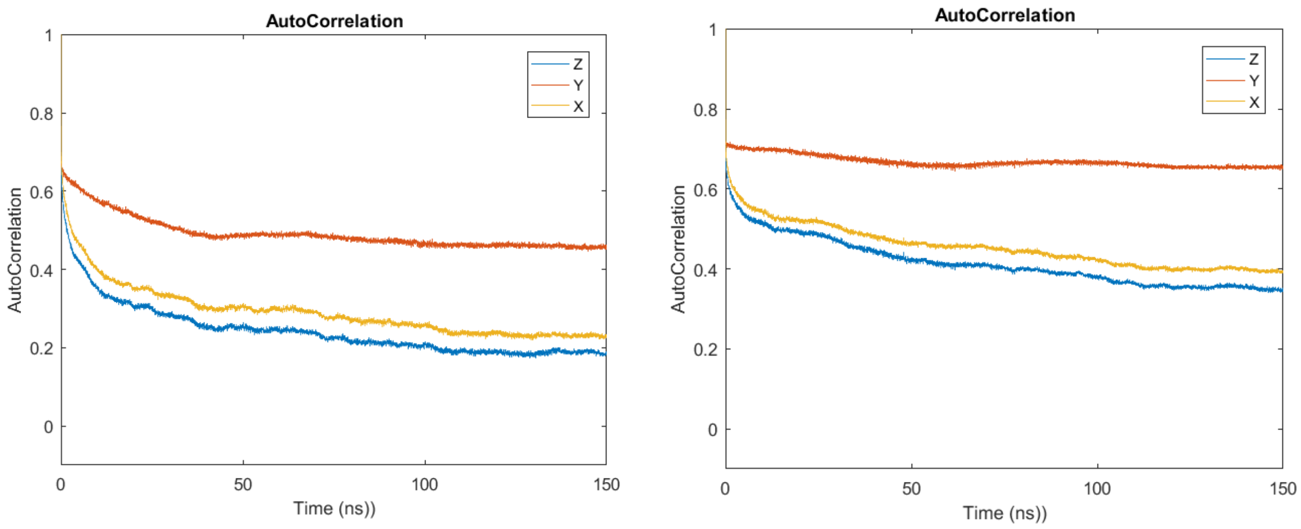

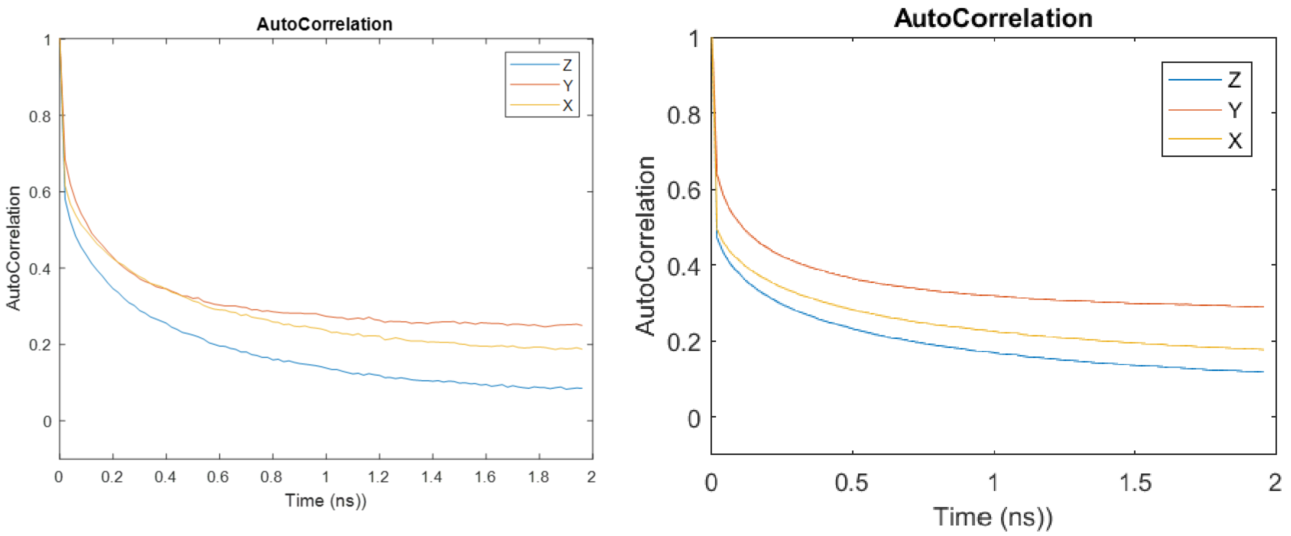


**Figure S10**. Example autocorrelation functions of solvent molecule fixed axes *x* (yellow), *y* (orange) and *z* (blue), calculated using Eq. (6) of the main text. (Top) FSPA acetone A (left) and B (right). (Bottom) FSPA ethanol A (left) and B (right). *z* is the unit vector along one of either the equivalent C–D or C–$CD_3$ axes in ethanol or acetone respectively, and *x* and *y* are mutually orthogonal axes in the plane perpendicular to *z*. Plots shown are for the 250 K simulations. Data for all temperatures can be found in the data archive.

**Table S5**. Magnitudes of principal components of the $^2$H quadrupolar tensor (in kHz and in increasing order) for model motions under fast motional averaging.

| | Two-fold flip | Rotational diffusion |
|---|---|---|
| Ethanol solvate | α = 109.5°<br>1.62<br>56.88<br>58.50 | α = 109.5°/2, β = 0<br>0.81<br>0.81<br>1.62 |
| Acetone solvate | α = 116.80°<br>3.52<br>19.96<br>23.48 | α = 116.80°/2, β = 0<br>1.76<br>1.76<br>3.52 |

**Table S6.** Effective correlation times and generalised order parameters for the *z* correlation functions obtained from MD simulations.

| *T* / K | Model A $\tau_{c,eff}$ / ns | Model A $S^2$ | Model B $\tau_{c,eff}$ / ns | Model B $S^2$ |
|---|---|---|---|---|
| **FSPA ethanol** | | | | |
| 350 | 0.031 | 0.14 | 0.025 | 0.078 |
| 300 | 0.062 | 0.19 | 0.052 | 0.098 |
| 250 | 0.229 | 0.19 | 0.188 | 0.177 |
| 200 | 4.112 | 0.192 | 7.051 | 0.175 |
| 150 | 27.76 | 0.39 | 9.78 | 0.45 |
| **FSPA acetone** | | | | |
| 350 | 2.76 | 0.002 | 1.45 | 0.051 |
| 300 | 11.57 | 0.080 | 10.93 | 0.27 |
| 250 | 11.66 | 0.23 | 15.42 | 0.39 |
| 200 | 13.24 | 0.548 | 11.73 | 0.648 |
| 150 | 23.18 | 0.50 | 6.27 | 0.748 |

## 6. Analysis of molecular dynamics simulations

**Table S7.** Computed average and RMSD densities (in kg m$^{-3}$) of the different simulation models averaged over 400 ns production trajectories at every 1 ps step.

| *T* / K | | ACE-A | ACE-B | ETH-A | ETH-B |
|---|---|---|---|---|---|
| **150** | Avg: | 1488.2 | 1480.4 | 1533.9 | 1531.3 |
| | RMSD: | 5.9 | 3.8 | 1.7 | 11.7 |
| **200** | Avg: | 1471.8 | 1467.7 | 1522.0 | 1517.1 |
| | RMSD: | 3.1 | 5.8 | 2.3 | 7.7 |
| **250** | Avg: | 1451.5 | 1458.5 | 1506.9 | 1501.5 |
| | Std. Dev: | 3.5 | 6.0 | 2.9 | 4.2 |
| **300** | Avg: | 1427.4 | 1439.6 | 1490.1 | 1491.7 |
| | Std. Dev: | 4.5 | 4.5 | 3.8 | 6.4 |
| **350** | Avg: | 1388.4 | 1413.5 | 1473.9 | 1472.7 |
| | Std. Dev: | 5.3 | 5.2 | 4.3 | 4.9 |

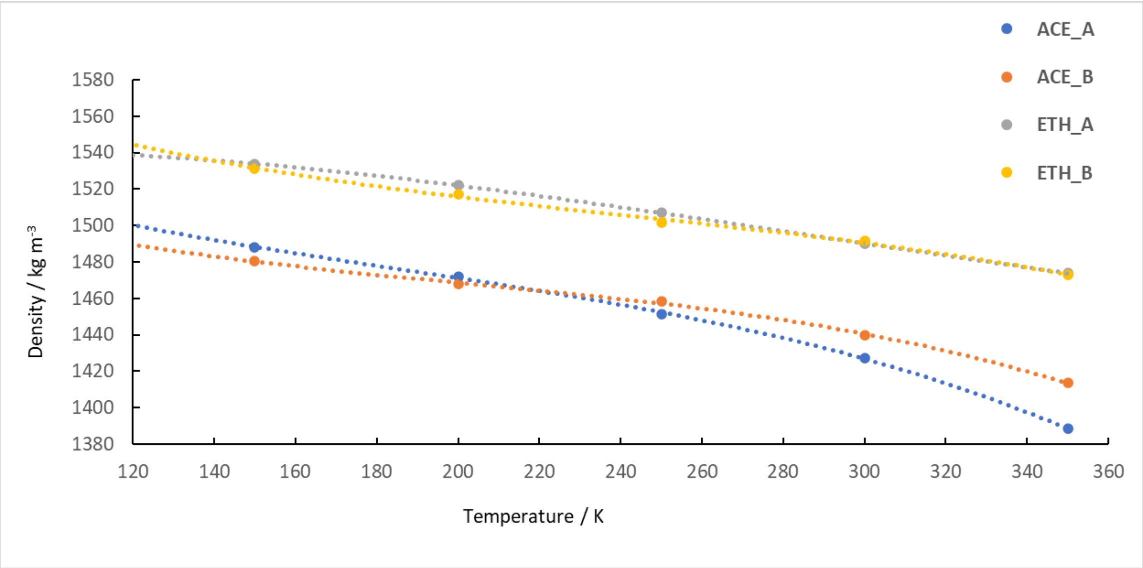


**Figure S11**. Change of density with temperature for FSPA acetone (ACE) and FSPA ethanol (ETH) systems, plus extrapolation to the experimental temperature (120 K) using fitting based on 3$^{rd}$ order polynomials. Data points are reported in **Table S8**.

**Table S8.** Computed (extrapolated) and experimental densities (in kg m$^{-3}$) at 120 K.

| | MD$^{a}$ | Experiment$^{b}$ | Deviation / % |
|---|---|---|---|
| FSPA acetone | 1490 | 1541.1 | –3.3 |
| FSPA ethanol | 1545 | 1465.3 | +5.4 |

$^{a}$Averaged over A and B models; $^{b}$Determined from the XRD crystal data as reported in **Table S1**, but including one molecule of solvent per unit cell. Note there is some uncertainty on the fraction of crystal sites that are occupied by solvent, whereas the simulations assume 100% occupancy.

## Analysis of sulfonamide conformation

Extended 400 ns trajectories were analysed to extract the dihedral angle patterns associated with the rotation of the sulfonamide groups on all FS molecules. The chosen dihedral angles along a trajectory were computed using the MDAnalysis tools[17]. Smoothed probability distributions of the dihedrals were computed using the Gaussian_KDE method from the Scipy package. The autocorrelation functions for the selected dihedral angles were computed using an approach similar to the one implemented in the GROMACS 'gmx angle' tool. Here, the dihedral autocorrelation function (dACF) is defined as[18]

$$C(t) = \langle \cos(\theta(\tau) - \theta(\tau + t)) \rangle_{\tau}$$

Here the periodicity issue is addressed by using the cosines rather than the dihedral angles themselves. In practice, to provide better statistics, the ACFs were calculated over multiple time origins while using data points with discrete time intervals Δ*t*, as follows,

$$C_f(j\Delta t) = \frac{1}{N-j} \sum_{i=0}^{N-1-j} f(i\Delta t) f\big((i+j)\Delta t\big)$$

where *N* is the number of available time frames in the trajectory. One should note that in this formulation all ACF points cannot be computed with the same accuracy, since there are *N*−1 data points for $C_f(\Delta t)$ time point, but only one data point for the $C_f\big((N-1)\Delta t\big)$ step. To avert the statistical biasing/inaccuracy arising from the latter, we compute only an ACF of length *MΔt*, where *M* ≤ *N*/2, which transform the ACF into the form

$$C_f(j\Delta t) = \frac{1}{M} \sum_{i=0}^{N-1-M} f(i\Delta t) f((i+j)\Delta t)$$

Here *M* is the time lag of the correlation function, which we took as *M* = *N*/2 for an equal representation of each data point in the set. The integral of the correlation function over time gives the correlation time $\tau_f$

$$\tau_f = \int_0^{\infty} C_f(t)\mathrm{d}t$$

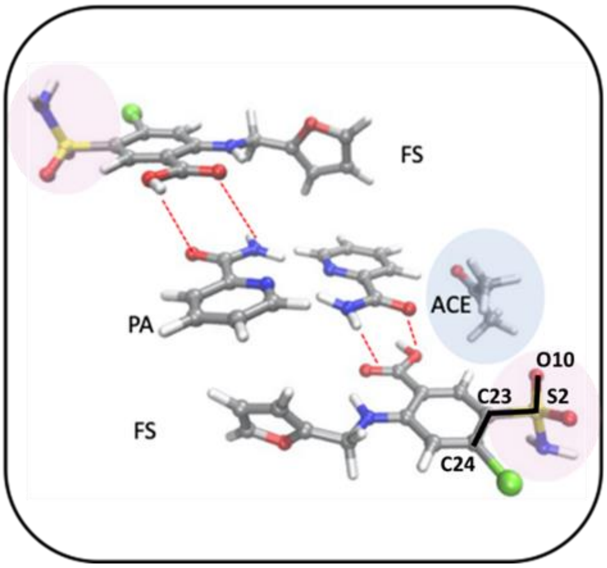


**Figure S12**. Atomic labelling of the sulfonamide group.

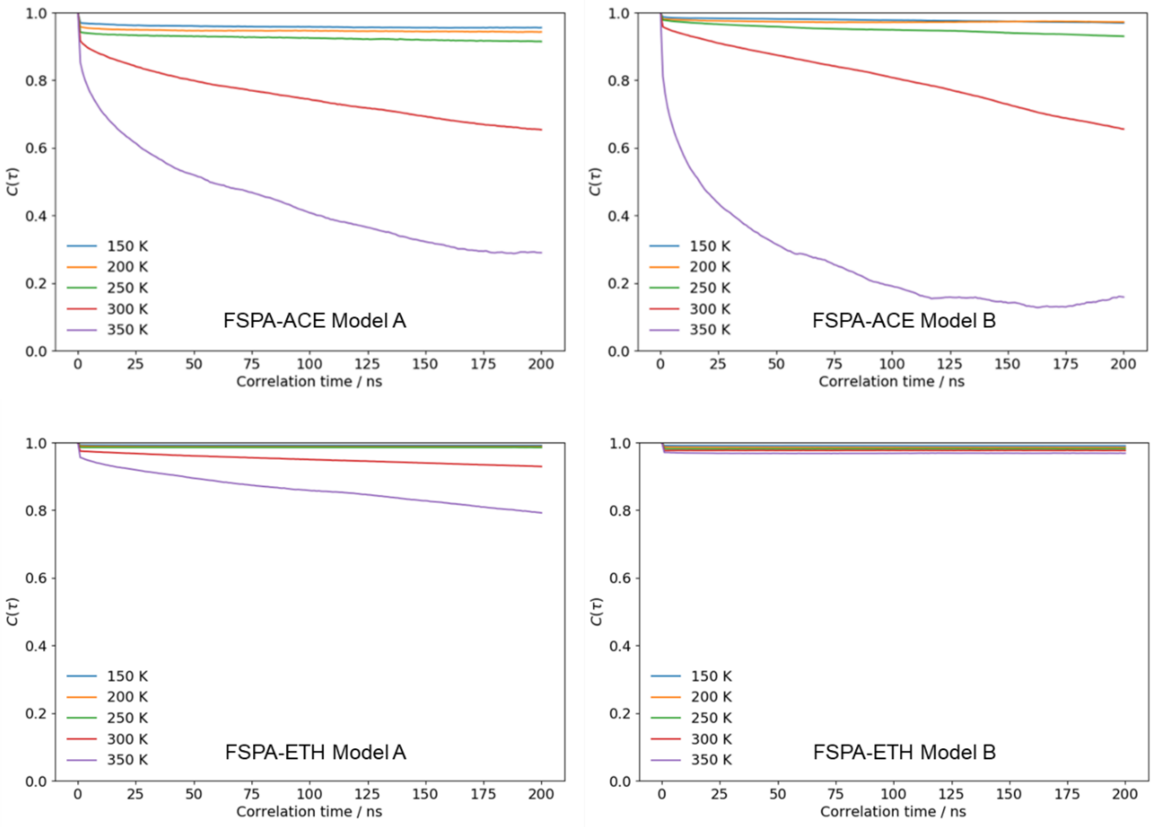


**Figure S13.** The autocorrelation function (ACF) of the dihedral angle, ϕ(O10-S2-C23-C24), describing the rotation of the sulfonamide groups with respect to the FS benzoyl, as a function of *T*. Atom indices are shown in Fig. S12.

**Figure S13** shows the autocorrelation function for the sulfonamide dihedral angle, ϕ(O10-S2-C23-C24). The results show that the sulfonamide rotation is thermally activated for the acetone system above 300 K, but orientations are mostly frozen below this temperature. Rotational motion of the sulfonamide group remains extremely slow for the ethanol system even at 350 K. The difficulty of sampling the sulfonamide disorder motivated our separate simulations of the idealised A and B systems. It is clear, however, that the sulfonamide is expected to be dynamic over the timescale of the experimental NMR.

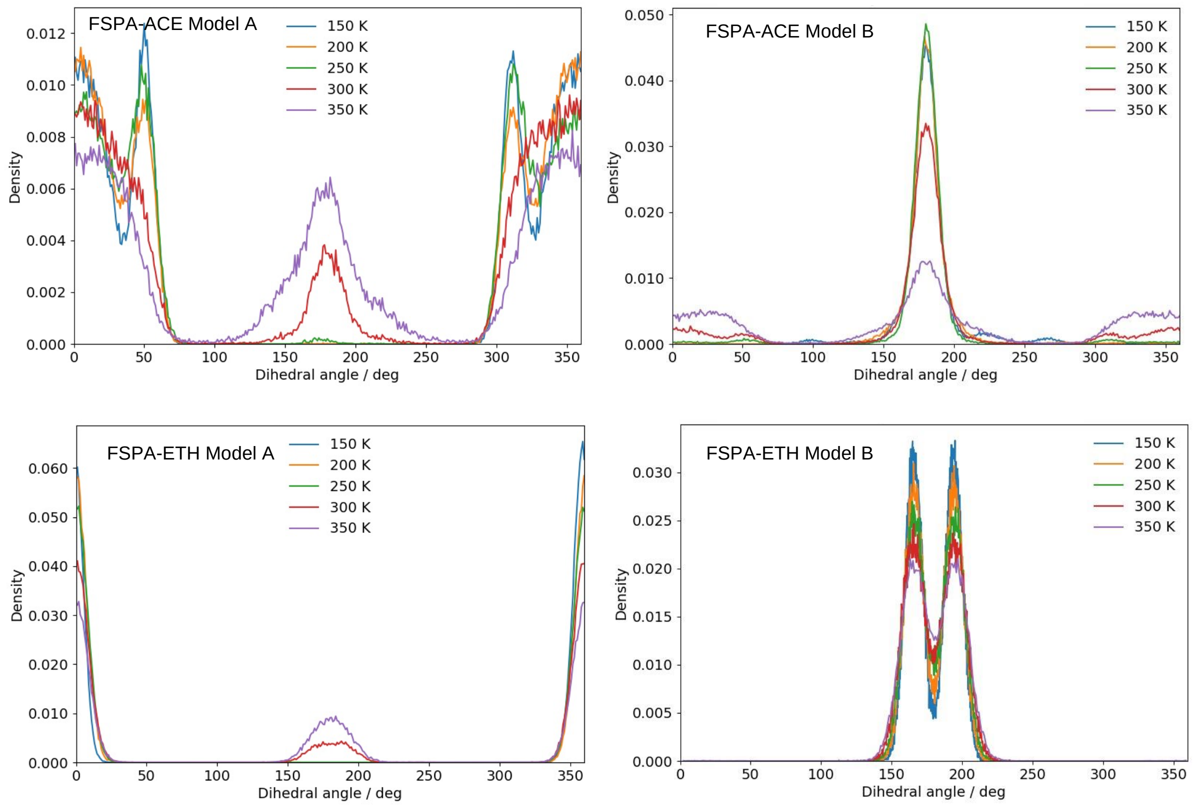


**Figure S14.** Probability density function/distribution of the ϕ(O10-S2-C23-C24) dihedral angle describing the orientation of the sulfonamide groups, as a function of *T*. Atom indices correspond to those in Fig. S12.

**Figure S14** shows the probability distributions for the ϕ(O10-S2-C23-C24) dihedral angle. At higher temperatures (*T* >= 300 K), the FSPA acetone simulations feature two distinct peaks indicating that the two orientations are increasingly equally populated with increasing temperature. In contrast, two double peaks (at 0° and 50° as well as at 300° and 350°) or two large unresolved peaks are observed below 300 K, depending on the input sulfonamide orientation (A or B). The latter indicates only a small, vibration-like movement of the sulfonamide groups rather than a full rotation (as in the high temperature cases). As for the effect of the initial sulfonamide orientation, there is a larger change in the probability distributions in model B than in model A in going from 150 K to 350 K. This corresponds to the larger ACF drop observed at higher temperatures, in Fig S13, particularly for model B.

For the FSPA ethanol system, on the other hand, we note only minor changes in the probability distribution of the dihedral angles compared to the acetone case because of very limited sulfonamide rotation (i.e. low amplitude motion rather than complete dihedral flips). Again, this is in line with the dihedral ACF trends.

## 7. Markov state modelling of MD trajectories

The Markov State Modelling was carried out using the PyEMMA[19] package. The protocol is described below, while the figures illustrating the key steps are visually summarised for the FSPA acetone and ethanol solvates in **Fig. S15** and **Fig. S16,** respectively.

**MSM analysis steps:**

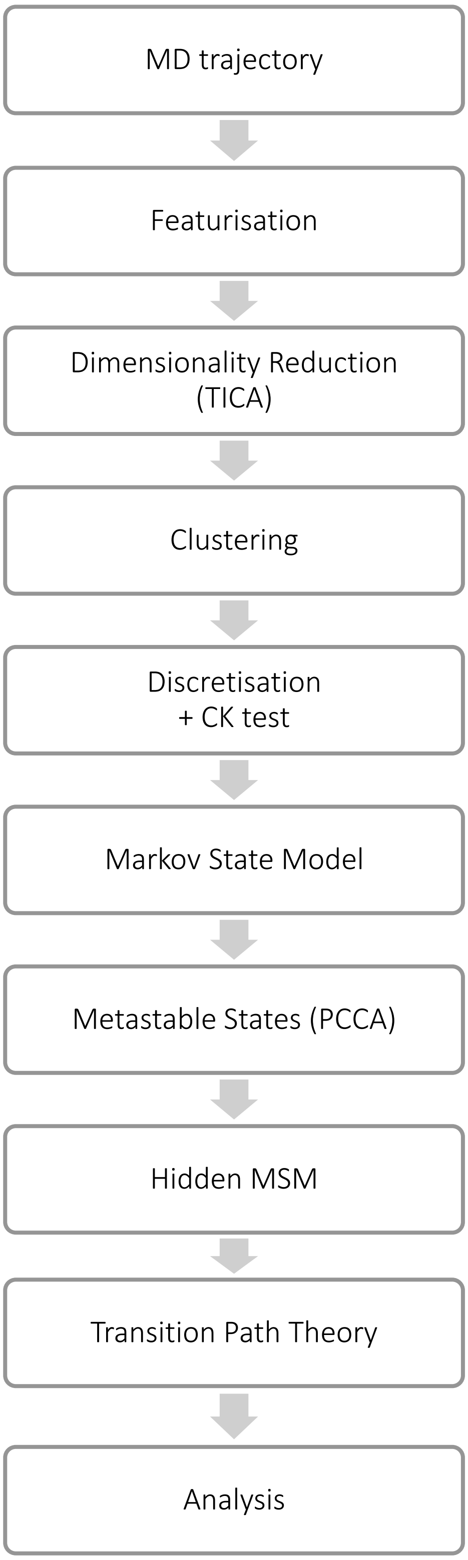


**MD trajectory**: changes in atomic positions with time, ‘continuous’ dynamics. Obtained from GROMACS simulations.

**Featurisation**: To minimise numerical calculation load, instead of using all atoms of the trajectory, we describe the system by a set of vectors (in spherical coordinates), see Fig S15(a), Fig16(a).

**Dimensionality Reduction**: TICA finds linear-combination with maximum auto-correlation time constant (i.e. slowest evolving) motion at a chosen lag-time. The lag-time estimate is shown as implied timescales (ITS) plot, Fig S15(b), S16(b). TICA is suitable for MSM, where kinetically slow processes are of interest. The first 4 TICs for each system are shown on Fig S15(c,d) and S16(c,d), note that those TICs represent over 98% of total motion in the system.

**Clustering** of TICA eigenspace to create a kinetically-relevant map of geometrical states. K-Means is a density-based algorithm. While it is possible to use a RegSpace (regular space) algorithm, it may lead to poorer sampling.

**Discretisation** of ‘continuous’ dynamics to create MSM. First, we assess how ITS are affected by the chosen lag time (and clustering). See Fig S15(b), Fig 16(b), where lines represent ITS (blue – slowest, followed by red and green) and shaded areas are lag-time propagation, i.e., motions that fall into this time cannot be resolved if a given lag-time is chosen. Second, we build a timescale separation plot, Fig S15(e). Since we aim to model transitions between macro-states, a clear separation is key (Fig S15(e)). We then carry out **Chapman-Kolmogorov** test to check for Markovian behaviour for the chosen number of the meta-states. If we are satisfied with the model, and no revisiting of the aforementioned steps is needed, we can confidently generate **Markov State Model** for our system.

**Metastable States**: Perron Cluster Cluster Analysis allows to define meta-states (i.e., long-lived states) based upon the created MSM. Unlike MSM, no hard cut-off states are used, so no dependence on the cluster-sizes, instead kernel distributions allow to account for the overlapping states, creating a **Hidden MSM** (Fig S15(f), S16(e)).

**Transition Path Theory** allows to extract highest flux pathways (Fig S15(e)) from the hidden MSM, which can then be used for any further **analysis** desired, such as identifying a pathway (Fig S15(h)) or determining temperature-dependant transition times (Tables S9, S10).

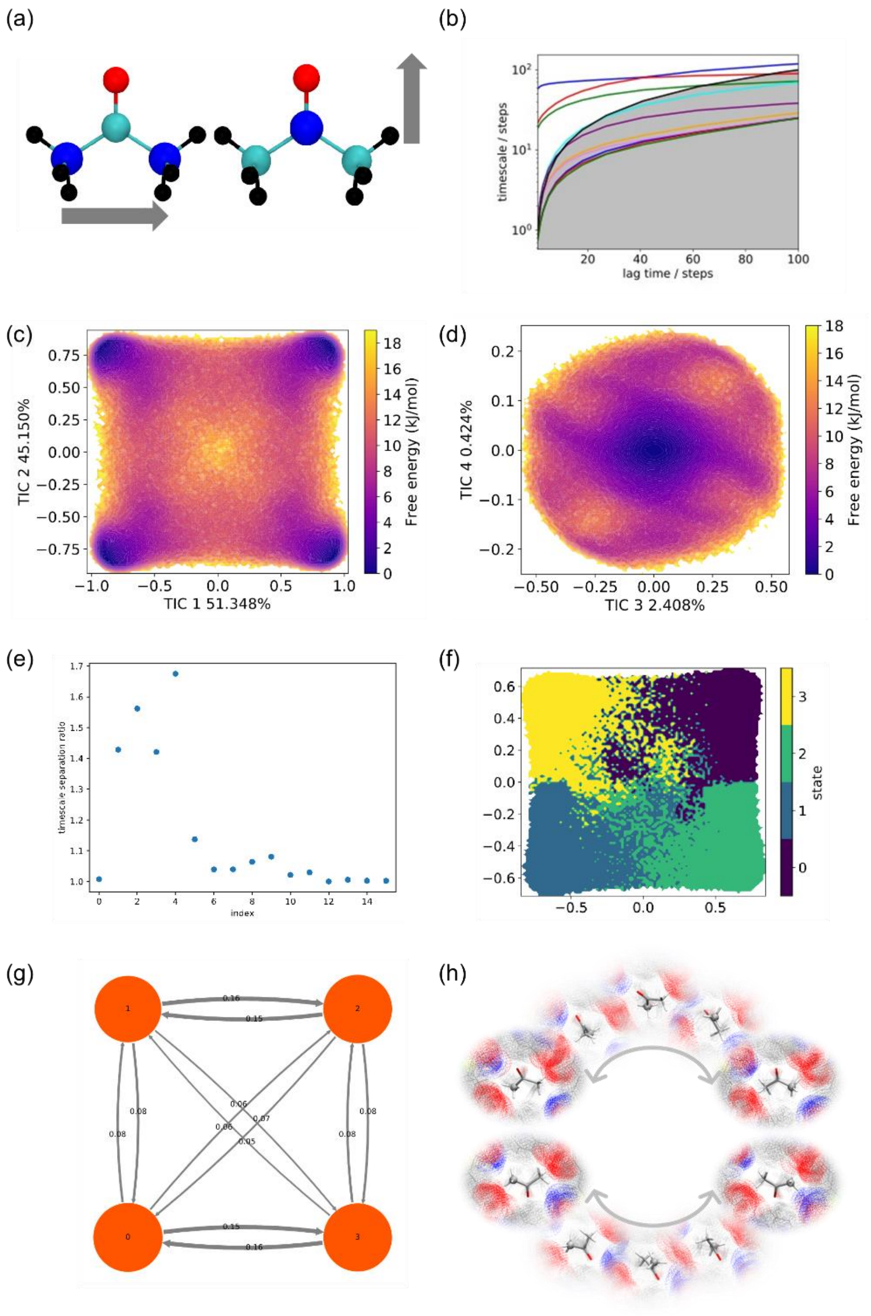


**Figure S15:** Development of MSM for the FSPA acetone system, using data from a 273 K simulation. (a) The choice of vectors to describe the acetone molecule. (b) Implied Time Scale (ITS) plot showing the dependence of each implied timescale on the MSM lag time (as a multiple of 0.5 fs timesteps). Free energy as a function of time-independent component (TICs): (c) TICs 1 vs 2, (d) TICs 3 vs 4. These graphs were produced using 256 cluster centres (microstates) and a lag time of 10 simulation steps. The fraction of the motion included in each TIC is given in the brackets. (e) Timescale separation plot, where each point shows the ratio of timescale $n$ and timescale $n + 1$, i.e. the first point shows the ratio between the first two timescales. (f) The division of the free energy diagram projected along TICs 1 and 2 into the four different "macrostates", denoted 0 to 3. (g) The state transition plot between these four macrostates. The size of each node (depicted in orange) is proportional to the steady-state probability, and thickness of the arrow is proportional to the transition probability (also given in numbers). (h) A pathway of molecular motion of the dominant flip, corresponding to the transition between nodes 1 ⟷ 2 and 0 ⟷ 3 in (g).

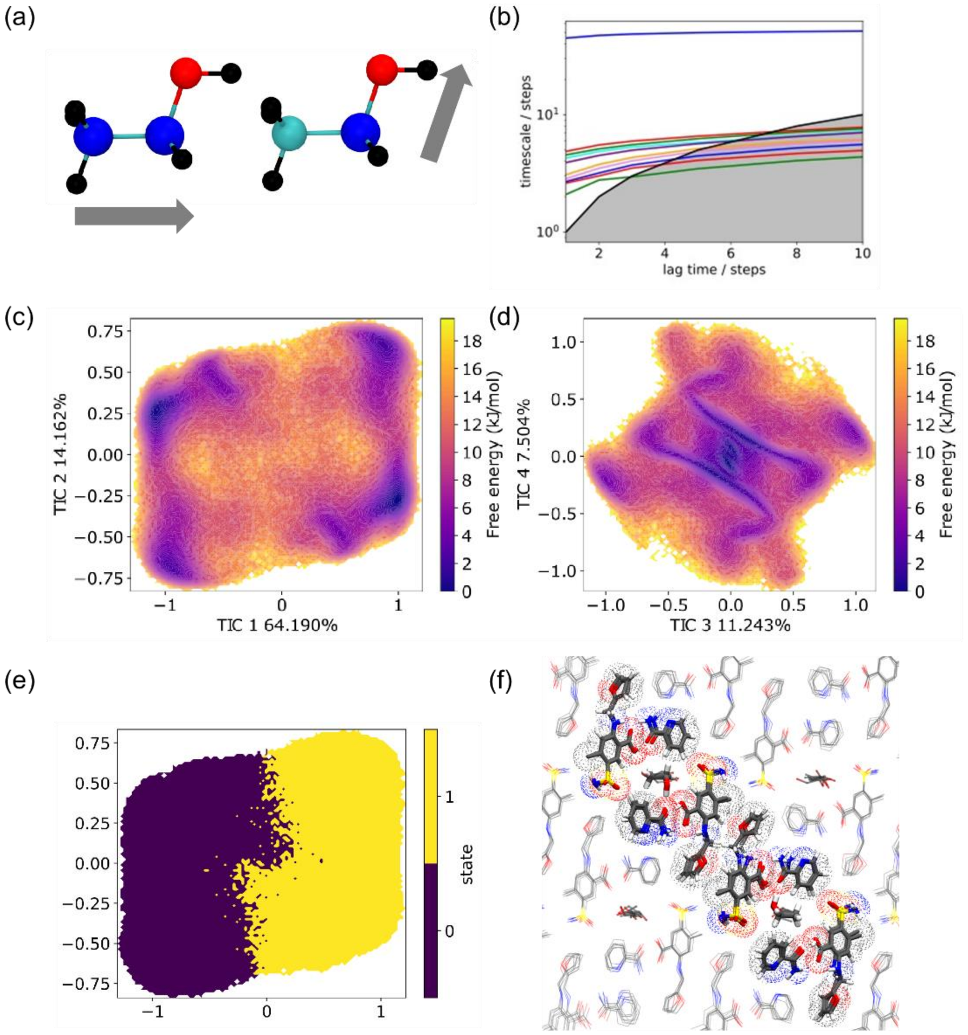


**Figure S16:** Development of MSM for FSPA ethanol system, using data from a 273 K simulation. (a) The choice of vectors to describe the ethanol molecule. (b) ITS plot. Free energy plots for (c) TICs 1 vs 2, and (d) TICs 3 vs 4, using 256 cluster centres (microstates) and a lag time of 10 simulation steps. (e) The division of the free energy diagram projected along TICs 1 and 2 into the two different metastable states. (f) Renderings of two states in a simulation.

**Table S9**: Average transition times between the two major states identified by MSM analysis for the FSPA ethanol system.

| Temperature / K | Average transition time / ns |
|---|---|
| 263 | 0.600 ± 0.007 |
| 273 | 0.560 ± 0.006 |
| 283 | 0.470 ± 0.005 |
| 294 | 0.362 ± 0.003 |
| 303 | 0.287 ± 0.002 |
| 313 | 0.219 ± 0.001 |
| 323 | 0.183 ± 0.001 |

**Table S10**: Average transition times between the four states identified by MSM analysis for the FSPA acetone system. The transitions correspond to the three paths between states seen in **Fig. S15 (g)**.

| Temperature / K | Average transition time / ns<br>0 ↔ 2 (1 ↔ 3)/ 1↔ 2 (0↔ 3)/ 0 ↔ 1 (2 ↔ 3) |
|---|---|
| 250 | 15.47 / 16.04 / 11.34 |
| 263 | 16.94 / 17.38 / 11.27 |
| 273 | 4.62 / 4.75 / 3.51 |
| 283 | 1.9 / 1.99 / 1.53 |
| 294 | 1.62 / 1.69/ 1.3 |
| 303 | 1.17 / 1.21 / 0.98 |
| 313 | 0.99 / 1.03 / 0.85 |
| 323 | 0.7 / 0.73 / 0.63 |

MSM analysis also allows transition times to be calculated between the major states identified in **Fig. 5**. **Tables S9** and **S10** summarise the average transition times obtained at each temperature for ethanol and acetone respectively. Fitting the temperature dependence to an Arrhenius equation yields an activation energy of 14.2 ± 0.4 kJ $mol^{-1}$ for ethanol and three activation energies, 24.9 ± 0.6, 24.8 ± 0.6 and 22.6 ± 0.6 kJ $mol^{-1}$ for acetone corresponding to the three identified pathways in **Table S10**. We note that the transition between the states 1 and 2 and between 0 and 3 states, corresponding to a rotation about the $CH_3$-$CH_3$ axis is generally slightly faster. Correlation analysis did not identify any significant correlation between the motion of neighbouring solvent molecules for either solvent.


1 H. M. Rietveld, *J. Appl. Crystallogr.*, 1969, **2**, 65–71.

2 A. A. Coelho, J. Evans, I. Evans, A. Kern and S. Parsons, *Powder Diffr.*, 2011, **26**, S22–S25.

3 B. I. Harriss, L. Vella-Zarb, C. Wilson and I. R. Evans, *Cryst. Growth Des.*, 2014, **14**, 783–791.

4 H. E. Kerr, L. K. Softley, K. Suresh, A. Nangia, P. Hodgkinson and I. R. Evans, *CrystEngComm*, 2015, **17**, 6707–6715.

5 H. E. Kerr, *NMR Crystallography of Disordered Cocrystals*, PhD Thesis, Durham University, 2017.

6 M. O. Miclaus, I. E. Kacso, F. A. Martin, L. David, M. M. Pop, C. Filip and X. Filip, *J. Pharm. Sci.*, 2015, **104**, 3782–3788.

7 V. S. Oganesyan, *Phys. Chem. Chem. Phys.*, 2011, **13**, 4724–4737.

8 V. S. Oganesyan, *Liq. Cryst.*, 2018, **45**, 2139–2157.

9 C. Prior, L. Danilane and V. S. Oganesyan, *Phys. Chem. Chem. Phys.*, 2018, **20**, 13461–13472.

10 SpinMolDyn, https://www.uea.ac.uk/groups-and-centres/projects/spinmoldyn.

11 G. Lipari and A. Szabo, *J. Am. Chem. Soc.*, 1982, **104**, 4559–4570.

12 M. Goldman, *J. Magn. Reson.*, 2001, **149**, 160–187.

13 B. M. Fung and T. W. McGaughy, *Biophys. J.*, 1979, **28**, 293–303.

14 R. L. Vold and G. L. Hoatson, *J. Magn. Reson.*, 2009, **198**, 57–72.

15 C. M. Gall, T. A. Cross, J. A. Diverdi and S. J. Opella, *Proc. Natl Acad. Sci. USA*, 1982, **79**, 101–105.

16 C. M. Gall, J. A. Diverdi and S. J. Opella, *J. Am. Chem. Soc.*, 1981, **103**, 5039–5043.

17 N. Michaud-Agrawal, E. J. Denning, T. B. Woolf and O. Beckstein, *J. Comput. Chem.*, 2011, **32**, 2319–2327.

18 D. Van Der Spoel and H. J. C. Berendsen, *Biophys. J.*, 1997, **72**, 2032–2041.

19 M. K. Scherer, B. Trendelkamp-Schroer, F. Paul, G. Pérez-Hernández, M. Hoffmann, N. Plattner, C. Wehmeyer, J.-H. Prinz and F. Noé, *J. Chem. Theory Comput.*, 2015, **11**, 5525–5542.

20 M. Banik, S. P. Gopi, S. Ganguly and G. R. Desiraju, *Cryst.Growth Des.*, 2016, 16, 5418-5428.
